# Should an R&D manager refer to distant technical fields? The effectiveness of new combinations with knowledge from different technical fields through the quantitative analysis of patent data related to NetZero

**DOI:** 10.3389/frma.2023.978249

**Published:** 2023-04-11

**Authors:** Masayuki Hirose

**Affiliations:** Ph.D. Program, Graduate School of Business Administration, Hitotsubashi University, Tokyo, Japan

**Keywords:** patent analytics, NetZero, distant knowledge, backward citations, technical field

## Abstract

This study showcases a technique to categorize NetZero-related patent applications into three technical fields according to the degree of proximity between claimed inventions and cited inventions by comparing technological classifications between the patent applications and cited applications thereof. In this technique, the author first describes the existing methods used in previous studies. The technique proposed in this article is different from those of previous studies in that it is characterized by comparing the technical fields of not only the primary classification but also the subsequent classifications. This is made possible by using two patent classifications without having a specific classification corresponding to the middle hierarchy in between, rather than using three patent classifications with different hierarchies. This technique reduces the possibility that two applications, even if they are the same in their subsequent classification, will be judged as applications in different technical fields because they are in different classes in the primary classification. Using the proposed technique, the author examined the impact on the subsequent patent application of NetZero-related patent applications filed in Japan. As a result of the analysis, the author found that approximately 33% of subject applications, whose technical field differs from the backward citations when comparing the primary classification only, match one of the subsequent classifications when comparing them in consideration of the subsequent classifications as well. The author then found that these 33% of subject applications had a greater impact on subsequent patent applications than the remaining applications.

## Introduction

This study showcases a technique to categorize NetZero-related patent applications into three technical fields according to the degree of proximity between claimed inventions and cited inventions by comparing technological classifications between the patent applications and cited applications thereof.

NetZero refers to a state in which the greenhouse gases including carbon dioxide going into the atmosphere are balanced by removal from the atmosphere. Achievement of NetZero is necessary to prevent further global warming because global warming is considered to be interlinked with climate change. In the 26th session of the Conference of the Parties to the United Nations Framework Convention on Climate Change (COP 26) held in November 2021, the participating countries proposed an action to reduce emissions.

The efforts for NetZero in Japan have already started 30 years ago. Fujii and Managi (2016) reported that more than 10,000 patent applications related to environmental protection technology have been filed every year since the 1990s with the increasing demands of the market and society for environmental protection and mitigation of climate change (Fujii and Managi, 2016, p. 4). Furthermore, the adoption of the Kyoto Protocol in 1997 spurred this trend of patent applications, and this trend seemed to continue. The OECD (2015) indicates that Japan has led the world in high-value invention in environmental technology (Fujii and Shirakawa, 2015, p. 3; Haščiči and Migotto, 2015, p. 27–35).

**Figure 1:**
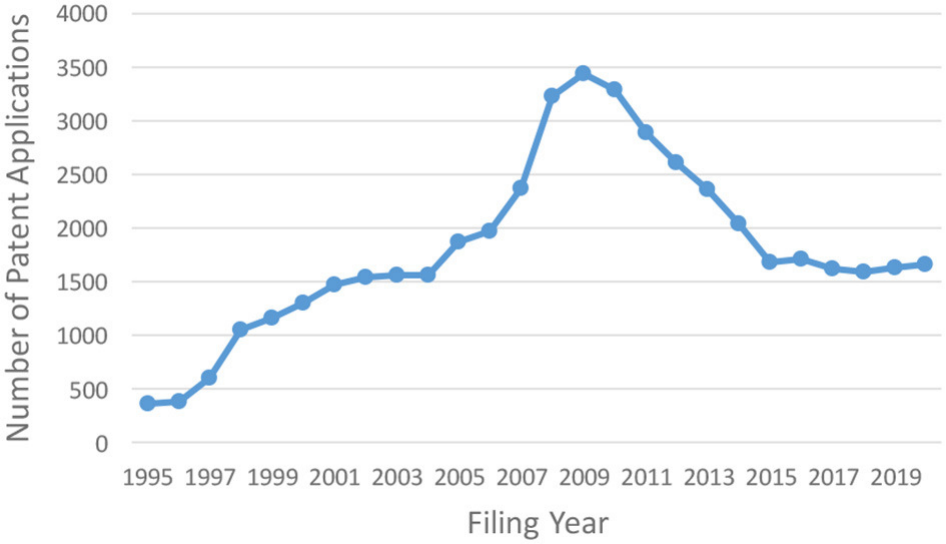
Changes in number of Japanese patent applications related to carbon neutral.

However, after 2009, when the global financial crisis arising from the bankruptcy of Lehman Brothers began to affect Japan, the number of Japanese patent applications gradually decreased until around 2015. Figure 1 is a graph showing changes in the number of Japanese patent applications in which “carbon neutral” and its related terms^1^ are described in the specification of patent applications filed over the 25 years from 1995 to 2020. As can be seen from this graph, the number of applications has declined since peaking around 2009 and has been almost flat since around 2015^2^. COP21 was held in 2021 against a background of international interest in environmental issues rising again. The Japanese government took this opportunity to declare in October 2020 that Japan would realize carbon neutrality by 2050, and, in April 2021, the government set a very ambitious target of reducing greenhouse gas emissions by 46% by 2030. Coping with the issue of global warming has become one of the most important tasks for companies as well. In Japan, the public and private sectors are currently working together to develop new technologies for NetZero. Achieving the goals set by the Paris Agreement will require innovation that brings more change.

To address this challenge, the author focuses on two long-standing concepts in innovation. One is that recombination is one of the processes that leads to new innovations (Shumpeter, 1934; Nelson and Winter, 1982; Hargadon and Sutton, 1997). Schumpeter identified five cases of new combinations.^3^ The other is that important innovation involves the transfer of knowledge from one technical field to another. Fleming (2001) suggested that new combinations of distant knowledge may give rise to a path-breaking innovation. Miller et al. (2007) stated that obtaining a remote source of knowledge is important to revitalize existing knowledge and develop new capabilities. There are many studies suggesting that the use of distant knowledge leads to the development of breakthrough inventions (e.g., Cohen and Levinthal, 1990; Katila and Ahuja, 2002).

By combining technological components from more distant technological domains, the degree of novelty goes up (Keijl et al., 2016). However, with a further increase in novelty through the inclusion of components from more distant technological domains, the familiarity of the invention will commonly decrease as compared with combining within the same technical domain and building on existing knowledge (Shane, 2000). Fu et al. (2013), who examined the effect of the distance of such analogical design stimuli as “near” and “far” on design solution generation, showed that if the stimuli are too distant, they can become harmful to the design process.

The use of “distant knowledge” has also been discussed in the context of corporate search in comparison to local knowledge (e.g., March, 1991; Rosenkopf and Almeida, 2003; Kim et al., 2013). Local search is defined as knowledge search in closely related, familiar, and similar technical areas, whereas distant search is defined as a search for knowledge across diverse technical areas beyond the familiar technical area (March and Simon, 1958; Nelson and Winter, 1982; Stuart and Podolny, 1996; Rosenkopf and Nerkar, 2001; Kim et al., 2013). Distant search, in contrast to local knowledge, makes it possible to acquire new and external knowledge from distant technical fields (Tushman, 1977).

On the other hand, it has also been pointed out that the accumulated capabilities of firms can limit the scope of searches and their ability to understand and apply new knowledge (Cohen and Levinthal, 1990; Miller et al., 2007). It has been also pointed out that barriers to the search and transfer of knowledge across organizational boundaries arise from the accumulated competencies of firms and the cognitive constraints of individuals (Walsh, 1995; Miller et al., 2007) and shared routines developed from the unique histories of organizations (Nelson and Winter, 1982; Leonard-Barton, 1992; Garud and Rappa, 1994, Tripsas and Gavetti, 2000).^4^

Furthermore, recently, in addition to the well-known concepts of incremental and radical innovations, there is an idea to focus on adjacent innovations between them. Nagji and Tuff (2012, p. 4) who proposed this idea mentioned that “adjacent innovation” involves leveraging something established firms do well into a new area. The “adjacent innovation” is considered to be in line with a series of movements that have been attracting attention in Japan in recent years, in which Companies cross industry boundaries and enter various fields. They try to cultivate new fields by taking advantage of their own strengths in different fields.^5^

To make such an innovation that addresses the issue of global warming and promotes NetZero, should R&D managers refer to a more distant technical field? (Research Question).

In tackling this research question, the author believed that it would be useful to consider the concept of “adjacent innovation” as well. In other words, the author thought it would be beneficial not only to divide a series of patent applications into two technical fields, namely, the “same” technical field and the “distant” technical field but also to consider and analyze the technical fields located in the “neighboring fields”.

With this in mind, the author looked at previous literature showing previous methods of categorizing patent applications into three technical fields. However, some common issues were found in the existing methods: since they focus on the primary patent classification when comparing the technical fields of backward citations, the problem remains that even if the primary patent classifications are different from each other, it is not possible to distinguish the possibility of matching one of the subsequent classifications. Therefore, to solve the problem raised earlier in this article, the author proposed a method of categorizing a set of patent applications into three technical fields according to the degree of proximity between claimed inventions and cited inventions (backward citations).

This article proceeds as follows. After reviewing the previous studies that categorize patent applications into three technical fields using the classification of cited applications (backward citations), the author addresses issues common to the previous methods in Section Previous literature. Next, after deriving a hypothesis in Section Hypothesis, the author showcases how the proposed technique works in Section Proposed measurement method, and describes the data and methods for analysis in Section Data and method for analysis. After testing the hypothesis in Section Results, the author discusses the analysis results in Section Discussion. Finally, the author presents the conclusion of this study in Section Conclusion with limitations and suggests future research directions.

## Previous literature

There is one natural way to examine the technological proximity between a patent and its citations by using the technical classifications listed on them, the value of which was suggested by Trajtenberg et al. (1997) and has been shown to be useful for assessing the nature and impact of inventions for patent applications (Fleming, 2001; Hall et al., 2001; Harrigan et al., 2017).

Trajtenberg et al. (1997) constructed a methodology for measuring the technical distance between a patent and its citations using a hierarchical structure of the U.S. patent classification system that consisted of three-digit patent classes, two-digit categories, and six very broad fields. Applying this methodology, they attempted to compare the “originality” of universities and companies. On the contrary, this methodology can be applied to indicate flows of knowledge from each cited patent to each focal patent. Next, the author focuses on previous studies that categorize the distance between the focal patent and its cited patents into three categories using backward citations and the patent classification. A representative study is conducted by Nemet and Johnson (2012).

### Previous literature on analyzing knowledge streams from three technology domains

Nemet and Johnson (2012) referred to each citation from a focal patent to a previous patent as a citation pair and measured knowledge flowing from one technical domain to another by comparing, for each pair, the technical classification assigned to each cited patent with classification for the citing patent. They axiomatically categorize each patent–citation pair into three categories by comparing their classifications at each level in the hierarchy. For example, a pair is coded as “near” if they are in the same sub-class; a pair is set as “internal” if they are in the same class; a pair is set as “external” if they are in different classes; and a pair is set as “far external” if they are in different super-classes. This coding scheme is used to develop three variables using counts of backward citation pairs for each patent: far external, external, and near (Nemet and Johnson, 2012, p. 13). They minimized truncation bias by imposing a 10-year window on both forward and backward citations so that each patent has a similar weight (Nemet and Johnson, 2012, p. 10).

The results of the measurements showed significant effect in the two most highly cited individual technology categories. They found that external citations had a significant negative effect on forward citations for the category of computers and communications (Nemet and Johnson, 2012, p. 26). They also found that external citations were much less effective than other types of citations for the category of medical and drugs. The coefficient for far external was not actually negative, but the ratio comparing it to other citations was very small (Nemet and Johnson, 2012, p. 26). Conversely, for these most highly cited individual technologies, citing technically closer prior art had a stronger effect than far citations (Nemet and Johnson, 2012, p. 28).

**Figure 2:**
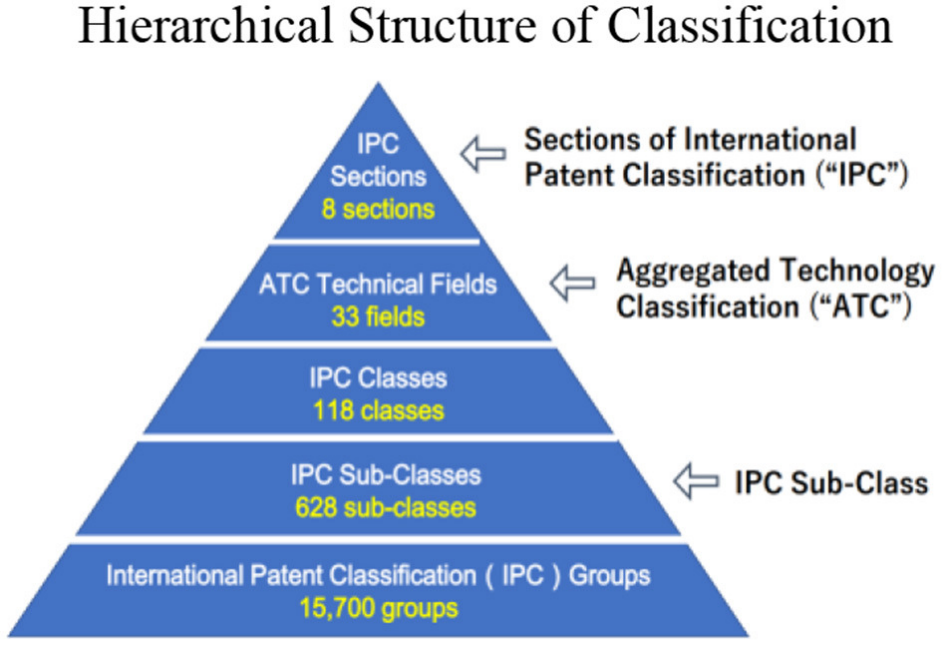
Hierarchical structure of classification.

Their explanation does not explicitly state that they used not only the primary classification but also the subsequent classifications. However, the phrase “our effort to use only high-level classes to compare results across multiple classification systems” (Nemet and Johnson, 2012, p. 30) is considered to imply that only primary classifications were used. They also count the total number of patents that the patent cites to define the citations made (Nemet and Johnson, 2012, p. 12). By using counts of backward citation pairs for each patent together with the abovementioned coding scheme, they developed three variables (far external *i*, external *i*, and near *i*) (Nemet and Johnson, 2012, p. 13). The counts of backward citation pairs are also used to compute the counts of other citations. Therefore, it is considered that the number of backward citations is taken into account in the development of these three variables. It is also understood, from Figure 2 and Table 1 provided by them, that “super-class” corresponds to “Section” in the case of the IPC. In addition, it is understood, from the coding scheme, that the pair coded as “far external” is used as the primary indicator of external knowledge flow and, thus, the IPC's eight sections correspond to “far external”.

**Table 1:** Top 50 NetZero-related technical fields.

**Categories**	**Nos**.	**Title**	**IPC subclasses**			
Energy creation technology	1	Fuel cells	H01M8	H01M4/86		
	2	Solar cells	H01L31	H01L51	H01M14	
	3	Wind motors	F03D			
	4	Nuclear physics; nuclear engineering	G21			
	5	Natural gas, liquid carbonaceous fuel	C10L	C10M	C10G	C10J
	C10K		C10B	F17C	
Processing, separation, mixing	6	Separation	B01D			
	7	Catalysis or colloid chemistry	B01J			
Material processing, laminate	8	Layered products	B32B			
	9	Shaping or joining of plastics	B29C			
Organic macromolecular compounds	10	Other than by reactions only involving unsaturated carbon bonds	C08G			
	11	Compositions of macromolecular compounds	C08L			
	12	Working-up; general processes of compounding	C08J			
	13	By reactions only involving carbon unsaturated bonds	C08F			
Organic chemistry	14	Heterocyclic compounds	C07D			
	17	Acyclic or carbocyclic compounds	C07C			
Biotechnology	15	Microorganisms or enzymes; compositions thereof	C12N	C12P		
Inorganic chemistry	16	Separation of hydrogen from mixtures containing thereof	C01B			
	18	Lime, magnesia, slag, cements, compositions thereof	C04B			
	19	Chemical composition of glasses, glazes or vitreous enamels	C03B	C03C		
Dyes	20	Coating compotitions	C09D	C09K3	C09K5	
Metallurgy	21	Alloys	C22C			
	22	Coating metallic material	C23C			
Heating (energy saving)	23	Fluid heaters, heat pump systems, heat-exchange apparatus	F24H	F25B	F28D	F28F
	24	Air-conditioning, air-humidification	F24F			
Energy storing technology	25	Secondary batteries	H01M10	H01M2	H01M4 (excluding 4/86)	
	26	System for supplying or distributing electric power	H02J			
	27	Containers for storage or transport of materials	B65D			
	46	Electrolysis: electrolytic or electrophoretic processes	C25B	C25C	C25D	C25F
Transporting technology	28	Controlling combustion engines	F02D	F02M		
	29	Control systems for hybrid vehicles	B60W	B60L	B60K	
	47	Traffic control system	G08G	G08B	G08C	
Conversion of electric power	30	Dynamo-electric machine	H02K			
	31	Control of electric motors/generators	H02P			
	32	Power converter	H02M			
Environmental aanalysis technology	33	Methods for supervisory or forecasting purposes	G06Q50	G06Q30	G06Q10	G06Q40
	34	Investigating or analyzing materials	G01N27	G01N21	G01N25	G01N30
			G01N33			
Optical technology	35	Optical elements or systems	G02B1	G02B5	G02B6	G02B26
			G02B27			
Display device	36	Circuit for control of indicating devices using static means	G09G			
	42	Display device for variable information	G09F9			
Printing technology	37	Photomechanical production of textured surfaces	G03F			
Environment technology	38	Treatment of wastewater or sludge	C02F			
	39	Disposal of solid waste, recovery from waste material	B09B	B09C	B29B	
	40	Clean and detoxify exhaust gas	F01N			
Energy saving technology	41	Electric heating; electric light source, especially organic EL	H05B33			
Greening technology	43	Agriculture, herbicide and pest control	A01G	A01M	A01N	
Building technology	45	Foundations; excavations; embankments	E02D	E02F	E21B	E21C
			E21D	E21F		
	48	General building constructions; walls	E04B	E04D	E04C	E04F
Textile and fibers	49	Natural or man-made threads or fibers; spinning	D01F	D06F	D06M	D21C
			D21H	D03D	D02G	
Others	44	Drive device transmission mechanism	F16H61	F16H45	F16H57	F16H1
			F16H25	F16H41	F16H63	
	50	Spraying or atomising: applying fluent material to surfaces	B05B	B05C	B05D	

Nemet (2012) used the same method of measurement as Nemet and Johnson (2012), but in contrast to them, he showed that for energy technologies the integration of technologically distant prior art (i.e., distant knowledge) has a stronger positive effect on knowledge flows than the integration of technologically near prior art (i.e., local knowledge). He found that coefficients on external citations were positive and significantly larger than those for other types of citations in the analysis of energy patents, whereas coefficients on citations to prior art that is technologically near have a negative effect on forward citation frequency (Nemet and Johnson, 2012, p. 1268). However, he found no significant difference between external citations and other citations in the analysis of all other patents including highly cited areas such as computers and medical patents, except for the category of electrical and electronics, which partially overlaps with energy technology (Nemet and Johnson, 2012, p. 1268). The energy technologies he targeted for patent analysis were defined according to the list of energy patent classes in Popp and Newell (2009, p. 1261).

Keijl et al. (2016) also measured the three knowledge flows from local, adjacent, and distant domains. They tracked recombination patterns in biotechnology patent applications by weighting the number of backward citations with their relative distance from a patent's focal domain. Although they showed that an intermediate level of recombination had the highest impact, the concept of “an intermediate level of recombination” they used is different from the concept of the combination with technologies in the neighboring fields referred to in this article. Rather, it should be noted that the concept refers to a combination of local and distant domains or a combination of near-adjacent and distant technologies (Keijl et al., 2016, p. 35). They argued that “an intermediate level of recombination” consisting of these combinations had the highest impact because it combines both novelty and familiarity (Keijl et al., 2016, p. 35, 36).

The technological distance between a sampled patent and its cited patents was determined as follows: when they are assigned to one of the three-digit biotechnology classes, this citation is categorized as a “near” citation. When the backward-cited patent is not assigned to a three-digit biotechnology class, but assigned to another class within “chemicals” or “drugs and medical”, both of which were some of the categories aggregated by Hall et al. (2001), this citation is categorized as an “adjacent” citation. When the backward-cited patent is not assigned to the “chemicals” or “drugs and medical” category, this citation is categorized as a “distant” citation. Next, the level of recombination was calculated by attaching different weights, respectively, to the three kinds of citations with near citations as “1”, adjacent citations as “2”, and distant citations as “3”. The sum of these weighted backward citations is then divided by the number of backward citations (Hall et al., 2001, p. 22).

They were aware that patents could be assigned to multiple patent classes, but only considered the number of patent classes as a controlling variable in their regression models. They controlled cross-reference classes other than the primary patent class used for the sampling strategy. This is because they viewed multiple patent classes as another possible indicator of the scope of a patent, and thus they considered that there was a possibility that patents assigned to multiple patent classes are cited by subsequent patents with a wider variety of patent classes. Therefore, this is different from the aim of this article to consider not only the primary classification but also the subsequent classifications of patents in order to analyze knowledge flows from other technical fields (Hall et al., 2001, p. 26).

### Need for modified measurement methods of patent content proximity

All of the previous studies discussed earlier have simultaneously considered multiple levels of technological aggregation. However, there is still room for further improvement in existing measurement methods when analyzing patent data using patent classification and citation information.

The issue is that when comparing the technical fields of backward citations, only the primary patent classification assigned to them was considered and not the subsequent listed patent classes. Even if the primary classifications are different, there are many backward citations that the subsequent classifications have in common. As previous methods focus on only the primary patent classifications assigned to patents, the challenge remains that two patents common to any of the subsequent classifications assigned to the patents cannot be distinguished even if the primary patent classifications are different.

After this problem was pointed out in other contexts by Thompson and Fox-Kean (2005)^6^ and Benner and Waldfogel (2008), subsequent studies began to consider the use of additional classifications beyond the primary classification in order to reduce this problem in other measurement contexts (e.g., McNamee, 2013; Aharonson and Schilling, 2016; Kuhn, 2017). This is a problem that could commonly occur in the International Patent Classification (“IPC”) as well as the U.S. patent classification (“UPC”). As technology has become more complicated, there are many patent applications in which the two inventions differ in the first classification but match in the subsequent classifications.

## Hypothesis

Here, the author would like to return to the research question posed in Section Introduction. To make such innovation that addresses the issue of global warming and promotes NetZero, should R&D managers refer to a more distant technical field as many existing studies emphasize?

The author believes that subsequent patent classifications, like the primary patent classification, have some impact on subsequent patent applications. Then, it is considered that the results of the analysis considering the subsequent patent classifications shift the peak from the distant field to the neighboring field compared to the results of using only the primary patent classifications. Therefore, the author sets the following hypothesis.

Hypothesis: There is a mountain-shaped relationship (an inverted U-shaped relationship) between the patent proximity between claimed invention and cited invention and the impact of the subject application on subsequent applications.

In the next section, the author describes the proposed technique of this article, which makes it possible to consider the subsequent patent classifications.

## Proposed measurement method

In this section, the author explains how the methodological challenges discussed in the first section are solved by the technique proposed in this article.

### Methodology for judging the incorporation of technologies from other fields using backward citations and patent classification

To identify patent applications for inventions incorporating knowledge in different technical fields, the author focuses on the possibility that “at least one” of the cited applications (backward citations) cited in the examination may be in a different technical field from the subject matter of the invention claimed in the subject patent application in the case that the invention incorporates technologies from other fields. This is based on the premise that the patent classification represents the subject matter of the claimed invention (Tsunoda, 2016, p. 267).

This is not limited to Japan but is commonly seen in patent examinations overseas as well. For example, Shane (2001) stated the following regarding the relationship between claimed inventions of U.S. patents and patent classifications assigned thereto:

“*The assignment of a patent to a particular patent class represents the U.S. Patent and Trademark Office's (USPTO's) assessment that the patent belongs in a particular technical field. Patent examiners also determine what previous inventions must be cited in a patent by searching prior patents. Because patents belong to technical classes and because they cite previous patents, citations to patents in particular technical fields represent the USPTO's assessment that a particular invention builds upon (cites) knowledge in that technical field. When a patent cites previous patents in classes other than the ones it is in, that pattern suggests that the invention builds upon different technical paradigms from the one in which it is applied.” (Shane*, *2001**, p. 210)*

What should be taken into consideration here is that inventions often consist of multiple technologies and that two or more patent classifications are often assigned to one patent application (Tsunoda, 2016, p. 266). Therefore, according to the proposed technique, all patent classifications assigned to the present application (including the primary classification and the subsequent classifications) are compared with all patent classifications assigned to its backward citations. Thus, it is checked whether there is “at least one” of the applications (backward citations) cited in the examination that is assigned a patent classification different from the patent classification assigned to the present application. If there is such a backward citation, the hierarchical structure of the patent classifications is used to categorize the degree of proximity between the present invention and the cited application into three levels: “same,” “neighboring,” and “distant”.

Furthermore, to characterize the proximities in the technological space defined by the classification scheme, the patent classification must be a ruler that reflects the state of the current technology and shows a hierarchical structure with an appropriate number of classifications between layers. To achieve this, the author proposes to combine two existing technical classifications, which are explained later.

### IPC-based aggregated technology classification

In the measurement technique proposed in this article, the Aggregated Technology Classification (“ATC”) based on the IPC is also used. This is described as one of the classification systems used in the WIPO statistical reports and matched to top-level categories in the NBER Patent Database with six technology types (Goto and Motohashi, 2005, p. 46; Goto and Motohashi, 2007, p. 1433). The IPC classification table is a set of classification items and has a structure in which all technical fields are arranged in a hierarchical, tree-like structure in different levels with codes, including sections, classes, and subclasses. In this article, the author proposes to use ATC, which does not have its own hierarchical structure, in combination with the IPC. There are two reasons for this. First, the ATC has an appropriate measurement range divided into 33 fields, as can be seen in Figure 2, which is narrower in its focus than the section level of the IPC with 8 sections and wider in its focus than the class level of the IPC with 118 classes. Second, the classification of the ATC is closer to the current state of technology than the IPC.

These reasons are supplemented with a comparative table (refer to Appendix I) that shows the relationship between the ATC and the IPC. In the table, the ATC technical field numbers (“ATC Nos”). One to Thirty-three are shown on the left, and the corresponding IPC sections and subclasses are shown on the right. In this table, pharmaceutical products (A61K), which are classified into IPC Section A representing Human Necessities together with Agricultural and Marine Products (A01) and pesticides/herbicides (A01N), are indistinguishable from them at the level of IPC section. In addition, enzymes (C12N9/) and genetic engineering (C12N15/) are classified together into the subclass C12N that represents microorganisms, and it is not possible to distinguish between the two at the subclass level. While genetic engineering is a state-of-the-art technology that is often subject to analysis, enzymes are essential technologies for food and medicine that produce Koji, yeast, and lactic-acid bacteria. Koji is the “national fungus of Japan” that is indispensable for fermenting sake, producing miso, soy sauce, and a whole range of traditional Japanese foods. In this way, the hierarchical structure of the IPC does not necessarily reflect the current state of the art in Japan.^7^ In contrast, the ATC has an independent classification for pharmaceuticals (No. 5) and separate classifications for biotechnology (No. 16) and genetic engineering (No. 17). Therefore, the ATC is more reflect the current state of technology than other classifications. It is neither too broad like IPC sections nor too detailed like IPC classes.

The proposed technique exemplifies the combination of the ATC and the IPC as classifications in different layers, but it is not limited to this combination. Rather, the feature to be emphasized is, instead of using three patent classifications with different hierarchies as in previous studies, the use of only two classifications consisting of the broadest patent class and the narrowest class. In the proposed technique, instead of using a specific classification corresponding to the middle layer, the target applications can be categorized into three technical fields by considering not only the primary classification but also the subsequent classifications. This point is a feature of the proposed technique. Although the author chooses the ATC and the ITC as the optimal combination for analyzing current technology in Japan, the combination can be changed depending on the times and the technology, and thus two classifications with appropriate spacing and hierarchical structure may be selected.

### Proposed measurement technique

Using the hierarchical structure of the primary classification and subsequent classifications given to patent applications, the author explains the proposed technique of categorizing a set of patent applications into three technical fields by comparing technological classifications between patent applications for an invention examined by an examiner (“Subject Application”) and prior applications cited by an examiner in the examination of subject application (“Backward Citations”).

#### Explanation of hypothetical examples

**Figure 3:**
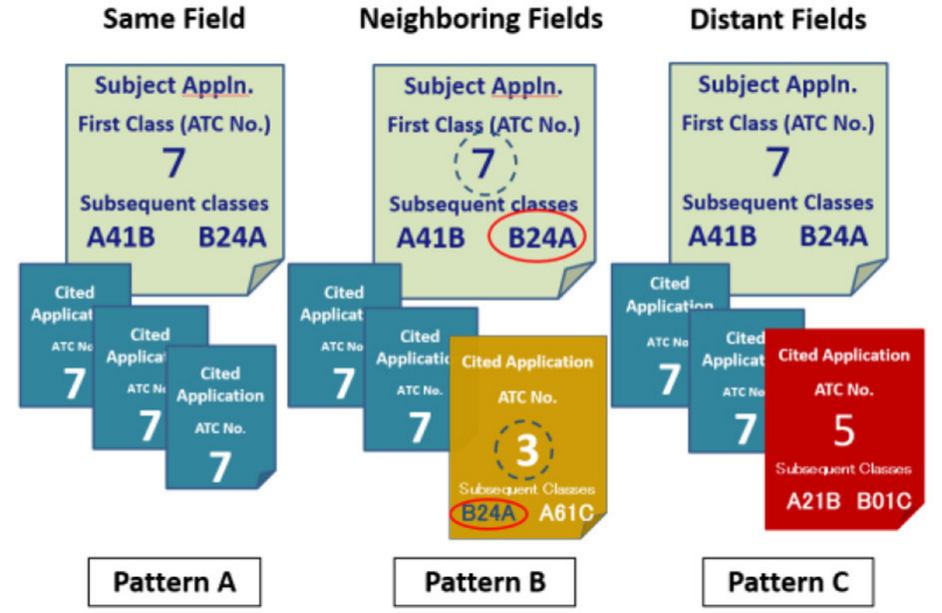
Three categories of subject applications depending on technical fields of backward citations.

Figure 3 shows hypothetical examples for explaining the measurement technique proposed in this article. In this figure, combinations of subject applications with three backward citations are shown as three patterns A–C. Each subject application is classified as ATC No. 7, which corresponds to the primary IPC classification assigned to each subject application. In contrast, the ATC classification of backward citations differs for each subject application. All backward citations of Pattern A are classified as the same ATC No. 7 as the subject application. For Patterns B and C, two of the three backward citations are classified as the same ATC No. 7 as subject application, but one backward citation is assigned a different ATC classification from its subject application.

The difference between the ATC No. 3 and No. 5 classification numbers does not make much sense here, and it is sufficient if they are different from the ATC classification of the subject application (No. 7 in this pattern). Rather, the point to be emphasized here is that in Pattern B, any one of the IPC subclasses of the backward citation (ATC No. 3 in this pattern) matches any of the IPC subclasses of subject application, whereas in Pattern C, the IPC subclass of backward citation (ATC No. 5 in this pattern) does not match any of the IPC subclasses of the subject application.

In summary, the differences between the three patterns are as follows. Pattern A is different from Patterns B and C in that the ATC Nos of all backward citations are the same as those of the subject application. Patterns B and C, in contrast, are similar in that at least one ATC No. of a backward citation differs from that of the subject application. However, Patterns B and C can be distinguished by comparing not only the primary classification but also the subsequent classifications.

**Figure 4:**
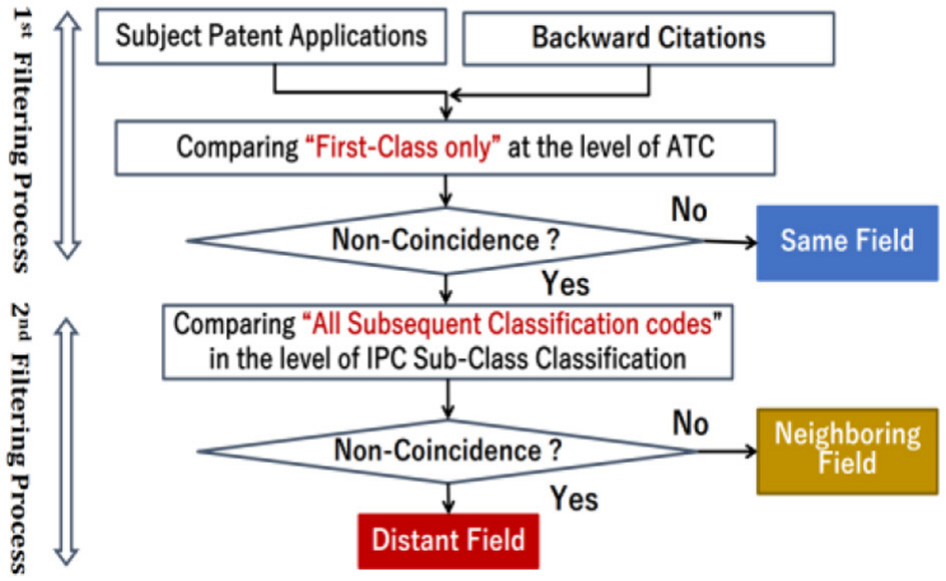
Flow chart of two-filtering processes.

According to the technique proposed in this article, the group data of a subject application can be categorized into the three patterns by comparing a subject application and its citations using two patent classifications with different hierarchies. Figure 4 shows the flowchart of the proposed technique. This figure shows a two-stage filtering process that compares a subject application and its backward citations using different hierarchies of patent classifications. The upper half of the figure shows the first filtering process using the ATC No. corresponding to the primary classification of the IPC subclass assigned in each application, and the lower half shows the second filtering process using subclasses of the IPC classification. In the second filtering, not only the primary classification but also all IPC classifications (including subsequent classifications) assigned to each application are targeted for comparison.

#### Extraction of subject applications falling into the same field

First, the first filtering process extracts subject applications one by one from a set of patent applications and compares subject applications and backward citations (usually multiple) with the ATC. Specifically, as shown in the upper half of Figure 4, comparisons are made using the ATC No. assigned to each application, and if they match (that is, if the judgment of “non-coincidence” is “No”), subject application is categorized into the Same Field. In contrast, if the comparison reveals at least one unmatched backward citation (that is, if the judgment of “non-coincidence” is “Yes”), analysis of the subject application can proceed to the second filtering process. This first filtering process may be done by analyzing with MySQL using the citation data of the IIP patent database.^8^

#### Distinguishing between subject applications falling into neighboring and distant fields

Next, the second filtering process is conducted for the set of applications extracted in the first filtering process. The second filtering process is performed, as shown in the lower half of Figure 4, by comparing “all IPC sub-classes including subsequent classes” assigned to the extracted subject applications and those assigned to backward citations. This is because, even if the subject application is different from backward citations at the level of the ATC, there is a possibility that the subject application has close proximity with any one of the backward citations at the level of the IPC subclass. In Pattern B of Figure 3, the subject application classified in ATC No. 7 differs from at least one other backward citation as far as its ATC No. is concerned. However, when compared with the IPC subclass of its backward citations, its subject application classified in IPC subclass B24A is consistent with one of its backward Citations.

To find such a combination of pattern B, if there is at least one of the backward citations that match the subject application at the level of the IPC subclass (that is, if the judgment of “non-coincidence” is “No”), the subject application is categorized into Neighboring Field. On the contrary, the subject application shown in Pattern C of Figure 3 is not only different from one of the backward citations categorized in ATC No. 5 but also different from its backward citation even when comparing with the IPC subclasses. The IPC subclasses A41B and B24A applied to this subject application do not match either of the IPC subclasses A21B and B01C applied to the backward citations. Thus, if there is no backward citation that matches the subject applications at the level of the IPC subclass (that is, if the judgment of “non-coincidence” is “Yes”), the subject application is categorized into Distant Field. This second filtering process is conducted using a commercial database with IPC patent classification data containing not only the primary classification but also subsequent classes of patent applications.

From the explanation mentioned earlier, it is understood that the proposed technique is different from those of previous studies in that it is characterized by (a) comparing the technical fields of not only the primary classification but also the subsequent classifications and (b) the use of the ATC having 33 classifications that are finer than IPC sections and broader than IPC classes. This is made possible by using two patent classifications without having a specific classification corresponding to the middle hierarchy in between, rather than using three patent classifications with different hierarchies. This proposed technique can be applied to compare not only between a subject application and backward citations but also between a subject application and forward citations (Hirose, 2021).

## Data and method for analysis

In this section, after describing datasets, the author explains how the proposed technique to identify patent “proximity” to test the hypothesis.

### Data

#### Preparation of preliminary data

To collect patent data related to inventions that promote NetZero, such as green technology and carbon neutrality, the author extracted the most frequently occurring natural languages and technical terms for NetZero from 5,249 articles published in the Nihon Keizai Shimbun from 2011 to 2021. Then, a search formula was created using the extracted terms to search for the Japanese patent applications filed in 2008.

The search formula is shown in Attachment II. As a result, 11,304 NetZero-related patent applications were extracted as preliminary data. In conducting the search, in order to prevent unintended search omissions due to the narrowing down of the IPC, the author avoided using patent classifications such as the IPC as much as possible. A method of searching a plurality of technical terms using proximity operators was adopted for the entire text of a patent specification. Therefore, it is undeniable that the search results may contain some level of noise, but a high priority was put on extracting inventions related to NetZero from a wide range of technical fields.

The preliminary data were grouped by IPC sections, and the grouped IPC sections were sorted in descending order of the number of patent applications filed in 2008. Therefore, IPC classes sorted in descending order of NetZero-related applications were prepared. Among the sorted IPC classes, those whose contents are related or close to each other are grouped together, and the top 50 NetZero-related technical fields are identified in descending order of the number of applications. Table 1 shows a list of the titles of the top 50 NetZero-related technical fields and the corresponding IPC classes.

#### Dataset of subject applications

Next, using the IPC classes corresponding to the top 50 NetZero-related technical fields, the patent applications that were filed in 2008 and have already been examined were searched. As a result, 67,302 patent applications with those IPCs in the first classification data were extracted. These patent applications were merged into one data table, including data on patent classifications, citation information, applicant information, and forward citations updated by March 26, 2022.

In this study, the analysis was carried out on Japanese patent applications filed in 2008. There are two reasons why this filing year was chosen. First, the year 2008 is considered to be the latest year for which the Japanese Patent Office has completed most of the examinations requested under the examination-on-demand system. Second, analyzing forward citations requires sufficient time necessary to measure the impact on subsequent applications. The filing year of 2008 refers to the actual filing year of the subject application. Thus, the filing year based on the priority date or the filing date of the patent application is not included. In the case of an international application, the year of the international filing date is adopted.

#### Dataset of backward citations

**Figure 5:**
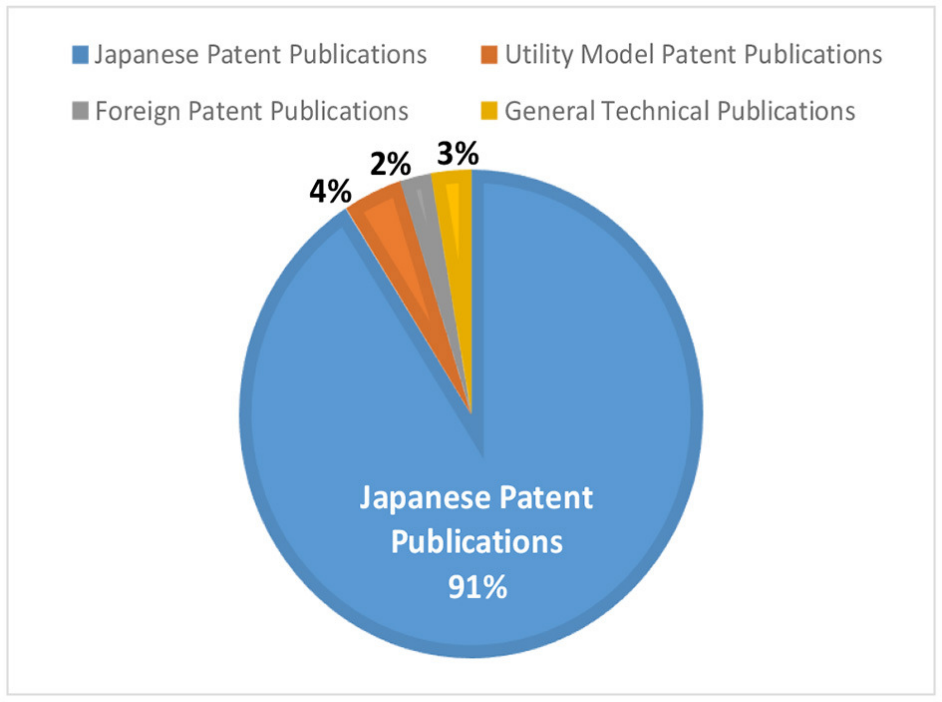
Percentage of publication types in citations.

The total number of backward citations in the 67,302 subject patent applications was 375,851. Among them, Japanese patent publications including international patent publications accounted for the largest share at 91%, and the remaining 9% were utility model patent publications (4%), general technical publications (3%), and foreign patent publications (2%), as can be seen in Figure 5. As utility model publications and foreign patent publications have a certain proportion of old scan data from the time before patent system was digitized, and general technical publications are not classified, they were excluded from this survey on the premise that it is considered in Section “Discussion”.

**Figure 6:**
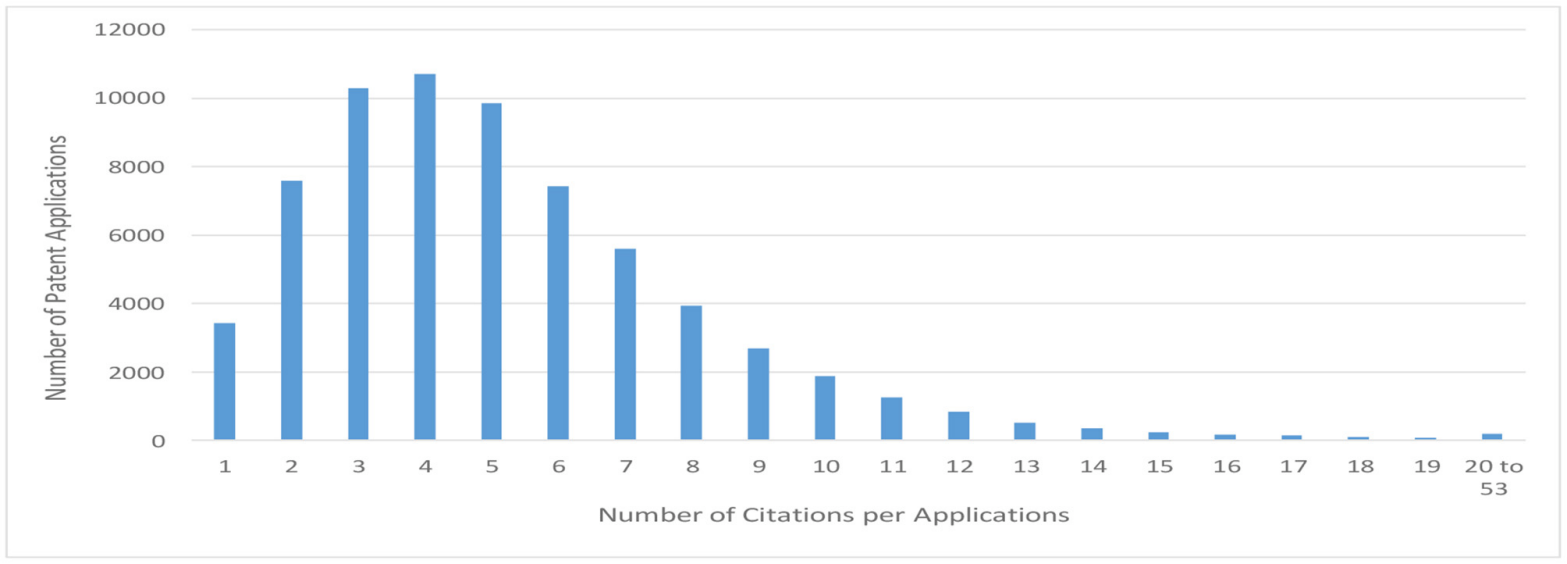
Bar graphs showing the distribution of the number of citations.

To reflect as much as possible the examiner's evaluation of the invention in this analysis, prior patent publications that are mentioned as citations in the official communication but are not cited as the basis for refusal are also included as backward citations in this study.^9^ On the contrary, the number of backward citations cited in a patent application varies from patent application to patent application, and the number reaches nearly 50 at most. It is not necessarily desirable, however, to easily increase the number of citations when considering the efficiency of analysis. Figure 6 is a graph showing the distribution of the number of citations. The horizontal axis indicates the number of citations per application in order of citation, and the vertical axis indicates the number of patent applications. With reference to this figure, the average number of citations per application in this survey is calculated to be six. Since the database used in this search records the citations in the order of description of the official actions, the citations mentioned by the examiner as the basis for refusal in the examination are recorded at the beginning, and the citations cited as reference tend to be recorded later. For this reason, the analysis in this study limited citations to six per application.

### Method for analysis

The author assumed that there is a mountain-shaped relationship (inverted U-shaped relationship) between the patent proximity of the claimed invention to the cited invention and the influence of the claimed invention on subsequent applications in Section Hypothesis. This means that a patent application in which a backward citation in Neighboring Field is cited in the examination is more cited in a subsequent examination than a patent application in which a backward citation in Distant Field or only the backward citation in Same Field is cited in the examination.

To test the hypothesis, the author uses the patent “proximity” between claimed invention and cited invention as an independent valuable and the impact of the subject application on subsequent applications as a dependent valuable. The proximity is represented by three technical fields consisting of “same,” “neighboring,” and “distant” according to the degree of matching of the technical fields of combined inventions using this proposed technique.

To investigate the impact of the subject application on subsequent applications, the author uses negative binomial regression for analysis of the value of the actual number of forward citations (“F-Citations”). This is because linear regression by ordinary least squares assumes that there is a linear relationship between the dependent and independent variables. However, in this analysis subjects, there is a possibility of non-linearity and, thus, it is necessary to deal with it. As a robustness check, the linear regression for the estimation of logarithm plus one of the dependent variable is performed. Furthermore, the number of claims in the patent application (“Claims”) and the number of pages in the patent specification (“Pages”) were added as control variables indicating the degree of disclosure in the specification.

In Section “Discussion”, the author compares the proposed method with Nemet (2012).

### Forward citations

To consider whether the invention will be a seed for future technological development, the authors investigate the impact of the invention on subsequent patent applications. A number of studies have focused on the technological impact of inventions as a way to assess their importance for technological development (Ahuja and Morris Lampert, 2001; Rosenkopf and Nerkar, 2001; Phene et al., 2006). The number of forward citations, widely used as an indicator of the value and quality of patents, is a value that indicates how much the patent is cited by subsequent patents, and the higher this value, the more influential the patent and the higher the value of the patent (Carpenter et al., 1981; Albert et al., 1991; Harhoff et al., 1999).

Regarding forward citations, there are “applicant citations” that the applicant describes in his patent specification and Information Disclosure Statement^10^ and “examiner citations” that the examiner cites as the basis for reasons for refusal when examining an application. Although there have been some skeptical opinions about the usefulness of the examiner's forward citation (Meyer, 2000; Mihara, 2012), it has been reported in the U.S. that “examiner citation” has a stronger influence on patent renewal than “applicant forward citation” (Hegde and Sampat, 2009; Cotropia et al., 2013; Yasukawa, 2016) or that the applicant's citation and the examiner's citation may not have been distinguished.^11^ In Japan as well, it has been reported that “examiner citation” correlates with the importance of patents, rather than “applicant citation” (Wada, 2010; Yamada, 2010; Yasukawa, 2016, 2017). Therefore, in this article that analyzes Japanese patent applications, “examiner forward citation” is used to investigate the effect of the invention on subsequent patent applications.

### Definition of technical terms pertinent to the model

In this article, the technical fields and the categories of subject application are defined as follows in relation to backward Citations:

Regarding patent applications and citations, “subject application” is defined as a patent application filed in 2008 and examined by the JPO and citing at least one patent application as prior art. “Backward citation” is defined as a prior patent application cited by an examiner in the examination of the Subject application.

Regarding the three technical fields, “Same Field” is defined as a technical field that is the same as that of the subject application in the ATC Nos. “Neighboring Field” is defined as a technical field that differs from the ATC No. of the subject application but is the same as at least one of the IPC subclasses given to the subject application. “Distant Field” is defined as a technical field that differs not only from the ATC No. of the subject application but also from any one of all IPC subclasses given to the subject application.

## Results

In this section, the hypothesis was tested for the dataset consisting of 67,302 patent applications (hereinafter referred to as “DATASET”) belonging to the top 50 NetZero-related technical fields.

### Results for extracting subject applications falling into same field

**Figure 7:**
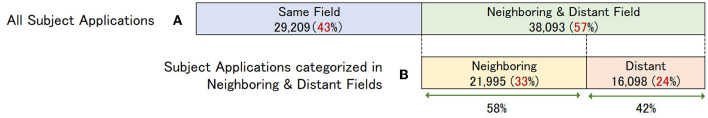
Graph showing the proportion of applications in the three technical fields in the total applications examined.

As a result of the first filtering process shown in the upper half of Figure 4, subject applications categorized in Same Field were separated from those categorized in Neighboring and Distant Fields. The number of subject applications categorized in Same Field reached 29,206, which accounts for 43% of the total applications of 67,302, whereas the number of subject applications in Neighboring and Distant Fields reached 38,093, which accounts for 57% of the total applications, as shown in Figure 7A.

### Results for extracting subject applications falling into neighboring and distant fields

Furthermore, as a result of the second filtering process shown in the lower half of Figure 4, subject applications categorized in Neighboring Field were separated from those categorized in Distant Fields. The number of subject applications categorized in Neighboring Field reached 21,995, which accounts for 33% of the total applications, whereas the number of subject applications in Distant Fields reached 16,098, which accounts for 24% of the total applications.

This means that, considering only the primary classification assigned to subject applications and their Cited Applications, the sum of Neighboring and Distant Fields accounts for 57% of the total applications examined shown in Figure 7A. On the contrary, Neighboring Field accounts for 58% of the 57% when considering subsequent classifications as well, as shown in Figure 7B.

### Advantages of including subsequent classifications

Table 2 shows the results of the subject applications categorized by the second filtering process, after being extracted by the first filtering process. The columns show the groups of citations in each application, which are shown in sets (Groups 1–6), in order of their citation, with up to six citations covered in this analysis. Results are shown for each of the six groups. The results are shown for Neighboring and Distant Fields according to the conditions shown in Figure 3 for each of the six groups. The point of focus here is the results indicated by the bold frame which take the subsequent classifications into consideration. For the result of non-coincidence when comparing only the first classification, it becomes possible to divide into Neighboring Field (26% of the total) and Distant Field (31% of the total) by adding subsequent classifications to the target of comparison. The number shown in the total column becomes the number shown in the “Remove Duplicates” column by subtracting the counted number of duplicates in multiple references to the same application.

**Table 2:** Table showing the results categorized by the second filtering process.

**Categories**	**Comparison of the subject patent application with each citation**		**Citations cited by the subject patent application**						**Total**	**Pct. (%)**	**Remove duplicates**
	**Between first classifications**	**Between subsequent classifications**	**Group 1**	**Group 2**	**Group 3**	**Group 4**	**Group 5**	**Group 6**			
Neighboring field	Coincidence (n^*^)		7,961	7,548	6,858	5,622	4,219	3,222	35,430	43	21,995
	Non-coincidence	Match at least one	4,683	4,693	4,118	3,469	2,687	1,940	21,590	26	
Distant field		None match	4,639	5,575	5,121	4,315	3,420	2,502	25,572	31	16,098
	Total	17,283	17,816	16,097	13,406	10,326	7,664	82,592	100	38,093

n^*^: The “coincidence” corresponds to one of citations, with one of the other five citations having a first classification of “non-coincidence”.

### Examples of patent applications categorized into three technical fields

Table 3 shows examples of patent applications categorized as “Same,” “Neighboring,” and “Distant” fields according to the proposed technique. The left-side columns show the patent applications (“subject applications”), and the right-side columns show the backward citations cited in the prosecution of subject applications (“backward citations”). The number of backward citations is listed in the order of citation of the examination and is limited to six. In each subject application and backward citation, the first classification in the given IPC sections and corresponding ATC Nos. are indicated by a bold border. To the right of the ATC column are all IPC subclasses assigned to each application and citation.

**Table 3:** Table showing examples of patent applications categorized into three technical fields according to the proposed technique.

		**Subject applications**					**Backward citations**				
	**No**.	**Application No**.	**Short title**	**IPC**	**ATC**	**IPC Sub-classes**	**No**.	**Application No**.	**IPC**	**ATC**	**IPC_Sub-classes**
Same field	1	2008-077964	Tungsten oxide photocatalyst	B	6	B01J, B01D	C1	1999-252707	B	31	B01J, A61L, B01D, B01J
							C2	1999-252707	B	31	B01J, A61L, B01D, B01J
	2	2008-312844	Variable valve timing controller for internal combustion engine	F	23	F02D	C1	2000-364588	F	23	F02D, F01L
							C2	2002-281495	F	23	F01L, F02D
							C3	2005-326262	F	23	F01L
	3	2008-230322	Lithium-ion storage device	H	31	H01M	C1	2005-259447	H	31	H01M
							C2	2003-131274	H	31	H01M
							C3	2003-419037	H	31	H01M
							C4	1998-179009	H	31	H01M
	4	2008-027941	Water heater using heat pump	F	25	F25B, F24F, F24H	C1	2004-303077	F	25	F25B
							C2	2007-049040	F	25	F25B, F24H
							C3	2000-184513	F	25	F25B
							C4	2000-184513	F	25	F25B
							C5	2004-025120	F	25	F25B
							C6	2001-024845	F	25	F25B
Neighboring fields	5	2008-022649	CO_2_ recovery apparatus	C	12	C01B, B01D	C1	2006-111302	C	12	C01B, B01D
							C2	1990-115393	B	6	B01D
							C3	1989-288559	B	6	B01D, C01B
							C4	2000-173353	B	6	B01D, C01B
							C5	1991-257666	B	6	B01D, C02F
							C6	2007-047421	B	6	B01D
	6	2008-020526	Power supply system using onboard storage battery	H	31	H02J, B01D	C1	1999-171641	H	31	H02J, B01D
							C2	2001-088042	B	10	B01D, G07F
							C3	1998-179111	B	10	B01D, B60S, G01R, G01V, G06F
							C4	2000-334844	G	28	G06F, B01D, B60S, H01M
							C5	2005-181781	H	31	H02J, B01D
	7	2008-058183	A method for regenerating an amine liquid	C	13	C07C, B01D, C02F, B01J	C1	1992-289047	B	6	B01J, B01D, C07C, C10G
							C2	1992-226969	C	13	C07C
							C3	2001-207991	B	6	B01J, C02F, F01K, G21C
							C4	1996-211190	C	12	C02F
							C5	2001-502951	B	6	B01D, B01J
							C6	1994-084916	C	13	C07C
Distant fields	8	2008-284960	An artificial algae bed construction	A	1	**A01G**, A01K	C1	2004-119188	A	1	A01K, B65D, C02F, E02B
							C2	1999-239179	A	1	A01K, B09B
							C3	1987-154283	E	21	E02B, E02D
							C4	1997-233809	E	21	E02B, A01G
							C5	2004-302144	A	1	A01K
							C6	2001-093906	A	1	A01K, A01G
	9	2008-184526	Charging system for electric vehicles	H	31	H02J	C1	1996-252494	H	31	H02J, B01D
							C2	1997-64927	B	10	B01D, B60K, B60R, H02J
							C3	1993-202726	H	31	H01R, B60K, B60R, H01M
							C4	1993-12448	G	28	G07F, H02B, H02J
							C5	1998-53923	G	28	G06F, G06K, G07F, G07G
							C6	1996-146433	G	28	G07C, G06F
	10	2008-109610	A power management system	G	28	G06Q, H02J, G06Q	C1	2002-224552	G	28	G08C, G01D, G06F, G08C
							C2	2001-281302	G	28	G05B, G06F
							C3	2000-358974	G	27	G01R, H02J
							C4	1998-147097	F	25	F24F
							C5	2006-271257	H	31	H02J, G01R, H02J
							C6	2007-019002	H	31	H02J, G01R

Yellow marking indicates that the IPC section differs between the subject application and the backward citation. Red marking indicates that the ATC No. differs between them.

Subject applications listed in rows Nos. 1–4 are examples of patent applications categorized as Same Field. A set of subject applications and backward citations that satisfy the conditions of Pattern A in Figure 3 is shown. This means that the ATC Nos. of all backward citations are the same as those of subject applications.

Subject applications listed in rows Nos. 5–7 are examples of patent applications categorized as Neighboring Fields. According to the conditions of Pattern B in Figures 3, 4, combinations of applications and citations satisfying the following two conditions are shown:

a) At least one ATC No. of a Cited Application differs from that of subject application.
b) In the combination that satisfies condition (a), there is at least one of the backward citations that matches subject application at the level of the IPC subclass (Corresponding IPC subclasses are indicated in blue). A clear example is given in row No. 6. In this case, there are two citations, C2 and C3 (Application Nos. 1998-53923 and 1998-145433), both of which have the same ATC number (No. 10). They are different from the ATC number (No. 31) of the subject application (Application No. 2008-020526), but they are common in the level of IPC subclass (B60L).

Subject applications listed in rows Nos. 8–10 are examples of patent applications categorized as Distant Fields. According to the conditions of Pattern C in Figures 3, 4, combinations of applications and citations satisfying the following two conditions are shown:

a) At least one ATC No. of a Cited Application differs from that of subject application.
b) In the combination that satisfies condition (a), there is no backward citation that matches subject application even at the IPC subclass level (Corresponding IPC subclasses are indicated in red). Row No. 9 provides a clear example of this. In this case, there are two citations, C5 and C6 (Application Nos. 1998-53923 and 1998-145433), both of which have the same ATC number (No. 28). They are different not only from the ATC number (No. 31) of the subject application (Application No. 2008-184526) but also from subject application in the level of their IPC subclasses.

In the previous examples, pay attention to the combinations of applications and citations determined to be Neighboring Fields (Corresponding IPC subclasses are indicated in blue). These combinations were determined to be “Neighboring” fields by considering all subsequent classifications into consideration. All of these examples are combinations that would be categorized as “Distant” fields if judged only with the first classification.

Nemet and Johnson (2012) allowed the use of the IPC in place of the U.S. System (Nemet and Johnson, 2012, p. 14). Even if IPC sections are used here, instead of ATC, as indicated by the black border in Table 3, the problems in Neighboring Fields are not resolved. A solution to this is to consider subsequent classifications, as emphasized in this proposal. This is the reason why the proposed technique is better than the ones in the previous method. The quantitative effect of using subsequent classifications is revisited in Section “Discussion”.

### Analysis of the entire dataset

Table 4 shows the result of negative binomial regression (“NB Regression”) analysis in assessing the effect of the combination with technology in Neighboring and Distant Fields on the actual number of forward citations (“F-Citations”). Table 5 shows the summary of the variables used therein. The evaluation results show that the NB Regression coefficient increases at the significance level of 1% or less as the technical field shifts from Same Field to Neighboring Field, whereas it decreases as the technical field shifts to Distant Field.

**Table 4:** Result of negative binomial regression.

	**(1)**
**Variables**	**Whole**
Neighbor	0.113^***^
	(0.0115)
Distant	−0.0135
	(0.0127)
Claims	0.00722^***^
	(0.000813)
Pages	0.00282^***^
	(0.000237)
Constant	0.632^***^
	(0.00986)
Observations	67,302
Pseudo *R*^2^	0.00198

Note: Standard error in parentheses, Significance level: ^***^p < 0.01, ^**^p < 0.05, ^*^p < 0.1.

**Table 5:** Summarizing variables of main analysis.

	**(1)**	**(2)**	**(3)**	**(4)**	**(5)**
**Variables**	***N***	**Mean**	***SD***	**Min**	**Max**
Pages	67,302	21.15	26.46	3	1,012
Claims	67,302	7.857	6.885	1	128
F_Citations	67,302	2.199	3.281	0	254
Neighbor	67,302	0.327	0.469	0	1
Distant	67,302	0.239	0.427	0	1

In Table 4, NB Regressions of Claims and Pages added as control valuables are also increased at a significant level of 1%. This is probably because the number of claims and the number of pages in the specification affect the degree of disclosure of the inventions. On the contrary, it is one order of magnitude smaller than the coefficients of Neighboring and Distant Fields, and the author believes that the influence of changes in the technological field is small.

**Figure 8:**
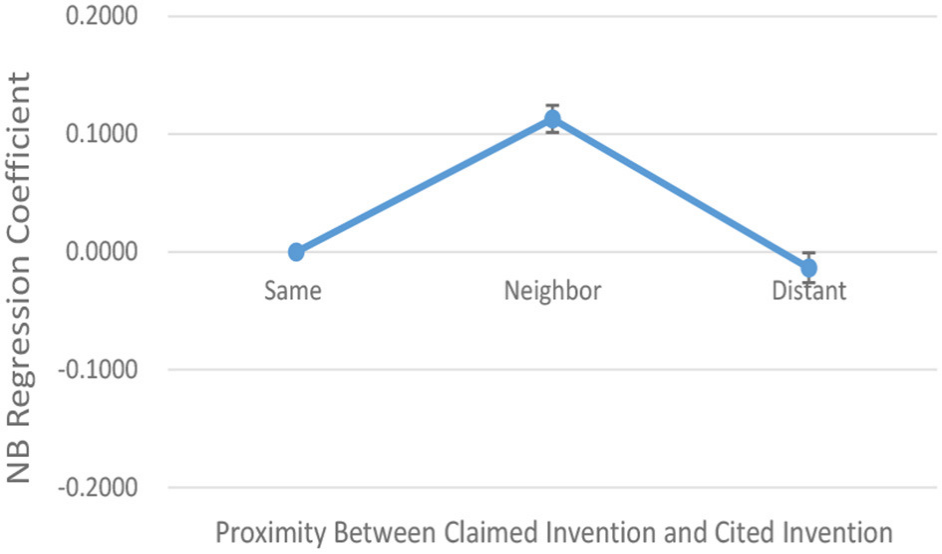
Graph showing the relation between NB regression coefficient and proximity with standard errors.

This result of regression analysis can also be seen in the line graph of Figure 8, which shows the relation between the NB Regression coefficient and proximity between claimed invention and cited invention. The proximity is shown as three technical fields consisting of Same, Neighboring, and Distant along the horizontal axis. The NB Regression coefficient is represented by the scale shown in the left vertical axis. As understood from the figure, the line graph of the NB Regression coefficient shows a mountain-shaped pattern, increasing as the technical field shifts from “Same Field” to “Neighboring Fields” and decreasing as the technical field shifts from “Neighboring Fields” to “Distant Fields”. Therefore, from the overall perspective of the DATASET, the Hypothesis is affirmed.

As a robustness check, the author also performed linear regressions for the estimation of logarithm plus one of the dependent variables (“F-Citations”). The results of this additional analysis are shown in Table 6. Table 7 shows the summary of the variables used therein. The comparison of the additional analysis with the main analysis shows that the results tend to be robust as the results of the former are substantially similar to those of the latter and significance levels remain stable.

**Table 6:** Result of ordinary least squares regression (OLS).

	**(1)**
**Variables**	**Whole**
Neighbor	0.258^***^
	(0.0293)
Distant	−0.0194
	(0.0321)
Claims	0.0152^***^
	(0.00195)
Pages	0.00557^***^
	(0.000508)
Constant	1.882^***^
	(0.0241)
Observations	67,302
*R* ^2^	0.006

Note: Standard error in parentheses, Significance level: ^***^p < 0.01, ^**^p < 0.05, ^*^p < 0.1.

**Table 7:** Summarizing variables of additional analysis.

	**(1)**	**(2)**	**(3)**	**(4)**	**(5)**
**Variables**	***N***	**Mean**	***SD***	**Min**	**Max**
Pages	67,302	21.15	26.46	3	1,012
Claims	67,302	7.857	6.885	1	128
F_Citations	67,302	2.199	3.281	0	254
Neighbor	67,302	0.327	0.469	0	1
Distant	67,302	0.239	0.427	0	1

### Analysis by technical field

Next, Hypothesis was tested using the NB Regression analysis on the DATASET divided by the top 50 technical fields to analyze the impact of the subject application on subsequent applications. The results of the NB Regression analysis are shown in Table 8. The summary of the variables used therein is shown in Appendix III. As understood from the comparison of the results for each of the fields of the IPC, the relationship between the results of the NB Regression analysis and the three technical fields shows unique tendencies for each subclass of the IPC. As a subject of verification, 25 fields in which the coefficient of either Neighboring or Distant Fields has a significance level of 10% or less were selected from Table 8.

**Table 8:** Tables showing the results of negative binominal regression for hypothesis.

	**No.1**	**No.2**	**No.3**	**No.4**	**No.5**	**No.6**	**No.7**	**No.8**	**No.9**	**No.10**
**Variables**	**H01M8 et al**.	**H01L31**	**F03D**	**G21**	**C10L et al**.	**B01D**	**B01J**	**B32B**	**B29C**	**C08G**
Neighbor	0.0223	0.0346	0.891^***^	0.146	−0.112	0.192^**^	0.148	0.120	0.203^***^	−0.120
	(0.0705)	(0.0829)	(0.274)	(0.151)	(0.0962)	(0.0778)	(0.0988)	(0.0966)	(0.0676)	(0.0748)
Distant	−0.0612	−0.0238	0.151	0.171	−0.0447	0.0870	−0.227^**^	−0.179^*^	0.184^**^	−0.105
	(0.0875)	(0.0835)	(0.243)	(0.158)	(0.0936)	(0.0981)	(0.112)	(0.0990)	(0.0729)	(0.0824)
Claims	−0.00362	0.00275	0.0199	0.0283^**^	−0.00681	0.00404	0.00292	−0.0124^**^	0.00172	−0.00503
	(0.00605)	(0.00462)	(0.0139)	(0.0124)	(0.00564)	(0.00581)	(0.00545)	(0.00603)	(0.00528)	(0.00499)
Pages	−0.00404	0.00222^*^	−0.00213	−0.00568	0.0151^***^	0.00541^*^	0.0153^***^	0.0201^***^	0.0178^***^	0.00759^***^
	(0.00362)	(0.00116)	(0.0172)	(0.00669)	(0.00403)	(0.00315)	(0.00369)	(0.00370)	(0.00374)	(0.00220)
Constant	0.748^***^	0.903^***^	0.0883	0.139	0.404^***^	0.368^***^	0.246^**^	0.709^***^	0.0912	0.759^***^
	(0.0692)	(0.0671)	(0.272)	(0.133)	(0.0815)	(0.0779)	(0.108)	(0.107)	(0.0703)	(0.0712)
Observations	1,703	1,400	132	411	1,090	1,295	1,150	1,423	2,157	1,464
Pseudo *R*^2^	0.000500	0.000931	0.0282	0.00565	0.00434	0.00266	0.0107	0.00847	0.00607	0.00256
**No.11**	**No.12**	**No.13**	**No.14**	**No.15**	**No.16**	**No.17**	**No.18**	**No.19**	**No.20**
**Variables**	**C08L**	**C08J**	**C08F**	**C07D**	**C12N et al**.	**C01B**	**C07C**	**C04B**	**C03B et al**.	**C09D et al**.
Neighbor	0.124^**^	0.175^*^	0.298^***^	0.228^***^	−0.0846	0.212^*^	0.0862	−0.00165	0.139	−0.0602
	(0.0578)	(0.0984)	(0.0893)	(0.0695)	(0.147)	(0.110)	(0.0896)	(0.0868)	(0.122)	(0.0886)
Distant	−0.0589	−0.178^*^	0.129	0.240^**^	0.0472	0.168	−0.00624	−0.245^**^	0.164	−0.222^**^
	(0.0647)	(0.100)	(0.0937)	(0.106)	(0.152)	(0.115)	(0.106)	(0.110)	(0.141)	(0.0897)
Claims	−0.00979^**^	0.00688	0.00893	0.000974	0.0105^*^	0.0114^*^	0.00921^**^	−0.00732	0.0215^**^	0.00670
	(0.00441)	(0.00695)	(0.00664)	(0.00283)	(0.00612)	(0.00612)	(0.00446)	(0.00745)	(0.00999)	(0.00520)
Pages	0.00994^***^	0.0139^***^	0.00956^***^	0.00352^***^	0.00388^**^	0.0145^***^	0.00326^***^	0.0163^***^	0.0297^***^	0.0173^***^
	(0.00225)	(0.00336)	(0.00226)	(0.000417)	(0.00194)	(0.00457)	(0.000765)	(0.00536)	(0.00848)	(0.00248)
Constant	0.810^***^	0.494^***^	0.363^***^	−0.0806	0.194	0.225^*^	0.160^*^	0.482^***^	0.0873	0.677^***^
	(0.0593)	(0.106)	(0.0897)	(0.0723)	(0.132)	(0.117)	(0.0822)	(0.0974)	(0.156)	(0.0931)
Observations	2,359	1,089	1,158	2,095	587	1,010	1,490	949	753	1,335
Pseudo *R*^2^	0.00284	0.00939	0.0102	0.0152	0.00657	0.00813	0.00758	0.00447	0.00983	0.0132
	**No.21**	**No.22**	**No.23**	**No.24**	**No.25**	**No.26**	**No.27**	**No.28**	**No.29**	**No.30**
**Variables**	**C22C**	**C23C**	**F24H et al.**	**F24F**	**H01M10**	**H02J**	**B65D**	**F02D et al.**	**B60W et al.**	**H02K**
Neighbor	−0.148^*^	0.137^*^	0.0293	0.0625	0.0841	0.454^***^	0.0708	−0.180^**^	0.277^***^	−0.105
	(0.0795)	(0.0772)	(0.0736)	(0.0790)	(0.0593)	(0.0816)	(0.0633)	(0.0802)	(0.0618)	(0.0787)
Distant	−0.328^*^	−0.0421	−0.0402	−0.0485	−0.0278	0.221^***^	−0.185^**^	−0.107	0.210^***^	−0.135
	(0.169)	(0.0881)	(0.0699)	(0.0696)	(0.0712)	(0.0857)	(0.0757)	(0.0894)	(0.0750)	(0.0828)
Claims	−0.00934	−0.00808	0.000855	0.0270^***^	0.0120^***^	−0.00646	−0.00562	0.00588	0.00572	0.0216^***^
	(0.00923)	(0.00571)	(0.00696)	(0.00765)	(0.00433)	(0.00557)	(0.00569)	(0.00748)	(0.00750)	(0.00699)
Pages	0.0150^**^	0.0182^***^	0.0121^***^	−0.00190	0.00760^***^	0.0330^***^	0.0209^***^	0.0210^***^	0.0166^***^	0.00617
	(0.00585)	(0.00408)	(0.00379)	(0.00373)	(0.00205)	(0.00463)	(0.00506)	(0.00435)	(0.00370)	(0.00425)
Constant	0.904^***^	0.321^***^	0.624^***^	0.656^***^	1.185^***^	0.737^***^	0.187^***^	0.177^**^	0.590^***^	0.526^***^
	(0.100)	(0.0877)	(0.0670)	(0.0722)	(0.0476)	(0.0875)	(0.0649)	(0.0753)	(0.0766)	(0.0676)
Observations	1,018	1,460	1,695	1,404	2,148	1,161	2,274	1,582	1,845	1,699
Pseudo *R*^2^	0.00333	0.00480	0.00214	0.00293	0.00379	0.0157	0.00362	0.00757	0.00635	0.00402
	**No.31**	**No.32**	**No.33**	**No.34**	**No.35**	**No.36**	**No.37**	**No.38**	**No.39**	**No.40**
**Variables**	**H02P**	**H02M**	**G06Q50 et al**.	**G01N27 et al**.	**G02B1 et al**.	**G09G**	**G03F**	**C02F**	**B09B et al**.	**F01N**
Neighbor	0.324^***^	0.0356	0.182^**^	0.0452	0.218^***^	0.173^**^	−0.117	0.0889	0.203	0.0398
	(0.0938)	(0.0862)	(0.0739)	(0.0537)	(0.0522)	(0.0812)	(0.0783)	(0.0843)	(0.171)	(0.108)
Distant	−0.215^**^	−0.107	0.0618	−0.137^***^	0.118^**^	−0.0782	−0.0851	−0.175	0.125	0.116
	(0.105)	(0.0912)	(0.0576)	(0.0532)	(0.0601)	(0.103)	(0.102)	(0.111)	(0.185)	(0.133)
Claims	0.00416	0.0133^*^	−0.000406	0.00505^*^	0.00230	0.0168^***^	0.0161^***^	−0.00279	0.00438	−0.00768
	(0.00870)	(0.00743)	(0.00334)	(0.00282)	(0.00439)	(0.00646)	(0.00623)	(0.00687)	(0.0134)	(0.00865)
Pages	0.0188^***^	0.00613	0.00524^***^	0.00549^***^	0.0140^***^	−0.00246	0.0115^***^	0.0205^***^	0.0192^**^	0.0164^**^
	(0.00666)	(0.00446)	(0.00199)	(0.00140)	(0.00185)	(0.00155)	(0.00137)	(0.00579)	(0.00805)	(0.00706)
Constant	0.351^***^	0.740^***^	0.712^***^	0.471^***^	0.431^***^	0.342^***^	0.542^***^	0.515^***^	−0.0649	0.620^***^
	(0.117)	(0.0755)	(0.0575)	(0.0433)	(0.0550)	(0.0905)	(0.0887)	(0.0982)	(0.187)	(0.119)
Observations	917	1,200	2,336	3,015	3,171	1,645	1,230	902	447	673
Pseudo *R*^2^	0.00972	0.00278	0.00149	0.00388	0.00785	0.00310	0.0171	0.00634	0.00684	0.00281
	**No.41**	**No.42**	**No.43**	**No.44**	**No.45**	**No.46**	**No.47**	**No.48**	**No.49**	**No.50**
**Variables**	**H05B33**	**G09F9**	**A01G et al**.	**F16H61 et al**.	**E02D et al**.	**C258 et al.**	**G08G et al.**	**E048 et al.**	**D01F et al**.	**B058 et al.**
Neighbor	−0.0208	1.161^***^	0.217^**^	0.225^**^	0.220^***^	0.0533	0.0573	−0.0624	0.361^***^	0.436^***^
	(0.113)	(0.277)	(0.0977)	(0.0888)	(0.0790)	(0.131)	(0.0874)	(0.0910)	(0.0767)	(0.127)
Distant	−0.0253	0.672^**^	0.292^***^	−0.0172	0.122	−0.331^**^	−0.00799	−0.128	0.282^***^	0.198
	(0.119)	(0.286)	(0.103)	(0.111)	(0.0839)	(0.152)	(0.0973)	(0.0842)	(0.0756)	(0.121)
Claims	0.00864	0.0188	0.0129^*^	0.00243	−0.0124	0.000284	0.00983	−0.00606	0.00975^**^	0.00124
	(0.00904)	(0.0133)	(0.00690)	(0.0107)	(0.00972)	(0.0105)	(0.00687)	(0.00986)	(0.00449)	(0.00929)
Pages	0.00693^*^	0.00151	0.00524^***^	0.0238^***^	0.0178^***^	0.0281^***^	0.0185^***^	0.0222^***^	0.0184^***^	0.0248^***^
	(0.00373)	(0.00756)	(0.00165)	(0.00541)	(0.00605)	(0.00868)	(0.00528)	(0.00569)	(0.00394)	(0.00640)
Constant	0.709^***^	−0.107	0.132^*^	−0.120	0.180^**^	0.116	0.646^***^	0.0543	−0.00797	−0.172
	(0.121)	(0.294)	(0.0788)	(0.110)	(0.0838)	(0.138)	(0.108)	(0.0759)	(0.0784)	(0.126)
Observations	632	606	1,006	1,026	1,422	479	979	1,703	1,673	904
Pseudo *R*^2^	0.00236	0.0106	0.00895	0.00864	0.00388	0.0126	0.00617	0.00373	0.0117	0.0125

Standard errors in parentheses.

^***^p < 0.01.

^**^p < 0.05.

^*^p < 0.1.

**Figure 9:**
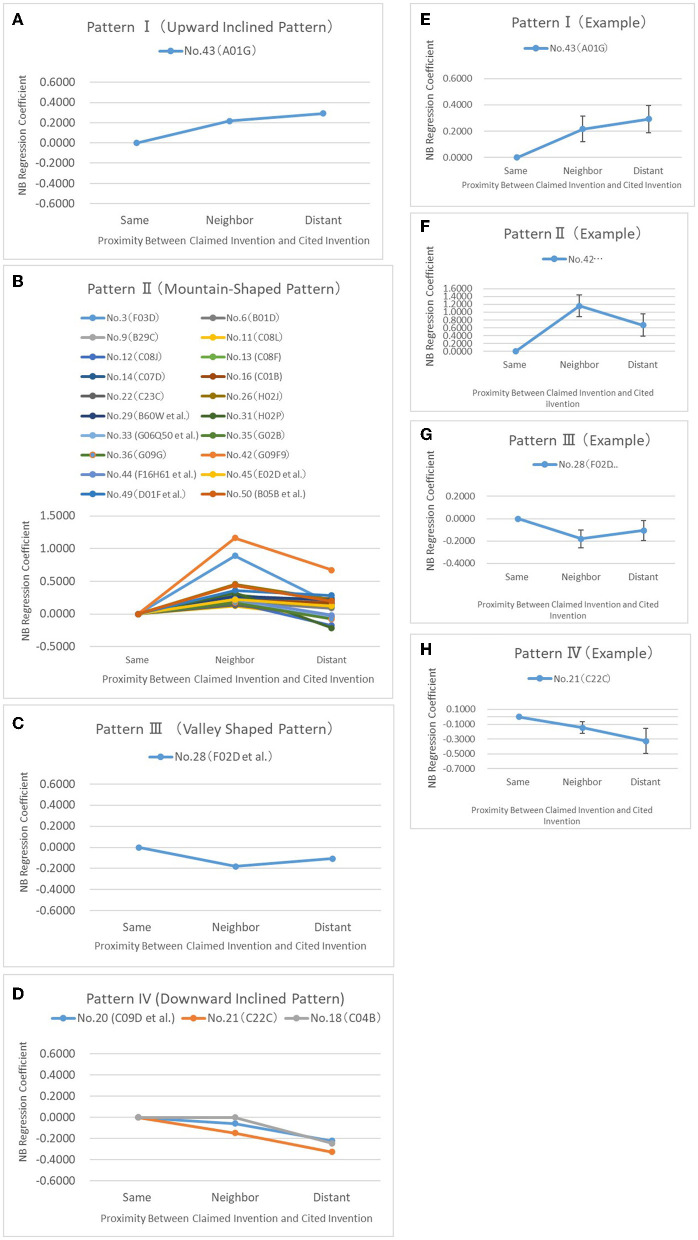
Graphs showing the relation between NB regression coefficient and proximity in selected 25 tecnical fields of top 50 for each of the four patterns.

Figures 9A–D are line graphs showing the change in the NB Regression coefficient for the 25 selected fields. They are grouped by four patterns I–IV depending on the technical fields of “Same Field” through “Distant Fields”: Pattern I shown in Figure 9A is the upward inclined pattern. Pattern II shown in Figure 9B is the mountain-shaped pattern. Pattern III shown in Figure 9C is the valley-shape pattern, and Pattern—shown in Figure 9D is the downward inclined pattern. Among these patterns, Pattern II corresponds to the technical fields that satisfy Hypothesis. From the comparison results for Patterns I through IV, it can be seen that there are many mountain-shaped patterns (Pattern II) in the analysis of the NB Regression coefficient. For example, the natural resins (C09F) listed as No. 42, as shown in Figure 9F, and the wind motors (F03D) listed as No. 3 indicate that the NB Regression coefficient increases at the significance level of 1% or less as the technical field shifts from Same Field to Neighboring Field but decreases as the technical field shifts from Neighboring Field to Distant field. Twenty ATCs, including these two, are shown in Figure 9B as technology fields that demonstrate the mountain-shaped pattern (Pattern II) that satisfies the hypothesis.

It can be seen that there is one example of an upward-inclined pattern (Pattern I) in Figure 9A. This is the cultivation of vegetable, rice, seaweed, forestry, and watering (A01G) listed as No. 43, which shows that the NB Regression coefficient increases at the significance level of 5% or less as the technical field shifts from Same Field to Distant Field, as shown in Figure 9E. This A01G was the only classification showing the upward-inclined pattern among the top 50 technical fields. As an example of the downward-inclined pattern (Pattern IV), Figure 9H shows the alloys (C22C) listed as No. 21 where the NB Regression coefficient decreases at the significance level of 10% or less as the technical field shifts from Neighboring Field to Distant Field. As an example of the valley-shaped pattern (Pattern III), Figure 9G shows the controlling combustion engines (F02D) listed as No. 28 where the NB Regression coefficient decreases at the significance level of 5% or less as the technical field shifts from Same Field to Neighboring Field.

To confirm the content of patent applications shown in the top 50 NetZero-related technical fields, the author reviewed several patent applications from them. A summary of 11 patent applications, taken as examples from six representative technical fields, is provided in Appendix V.

There are two reasons for analyzing the results by patent classifications that represent the technical fields. First, when conducting empirical studies using patent information, it is generally agreed upon to take into account that different technical fields have different views on the value of patents. It is generally agreed upon to take into account that different technical fields have different views on the value of patents (Mihara, 2012, p. 740). This is because there are technological fields such as chemistry and biotechnology where a small number of patents determine value and fields such as software and semiconductors where thousands of patents are intricately intertwined with a single product (Mihara, 2012, p. 740). Second, it is for comparing the method proposed in this article with those of the three previous studies dealt with in the first section. Nemet and Johnson (2012) mainly dealt with technical fields related to electricity and pharmaceuticals. Keijl et al. (2016) relied on the biopharmaceutical industry, and Nemet (2012) concerned energy technology, all of which target different technical fields.

### Analysis by IPC sections

Furthermore, Hypothesis was tested using the NB Regression analysis on the datasets divided into eight sections of IPC to analyze the impact of the subject application on subsequent applications. The results of the NB Regression analysis are shown in Table 9. Table 10 shows the summary of the variables used therein. As understood from the comparison of the results for each section of the IPC, the relationship between the result of the NB Regression and the three technical fields shows unique tendencies for each section. It can be seen from the figure that IPC Sections B (performing operations and transporting), C (chemistry and metallurgy), D (textiles and paper), G (physics), and H (electricity) demonstrate the mountain-shaped pattern (Pattern II) that satisfies the hypothesis. They show that the NB Regression coefficient increases as the technical field shifts from Same Field to Neighboring Field at the significance level of 1% or less but decreases as the technical field shifts from Neighboring Field to Distant Field.

**Table 9:** Result of negative binomial regression for IPC sections.

	**(1)**	**(2)**	**(3)**	**(4)**	**(5)**	**(6)**	**(7)**	**(8)**
**Variables**	**Best50_A**	**Best50_B**	**Best50_C**	**Best50_D**	**Best50_E**	**Best50_F**	**Best50_G**	**Best50_H**
Neighbor	0.217^**^	0.264^***^	0.0668^***^	0.361^***^	0.112^*^	0.0458	0.173^***^	0.108^***^
	(0.0977)	(0.0281)	(0.0219)	(0.0767)	(0.0594)	(0.0367)	(0.0258)	(0.0286)
Distant	0.292^***^	0.0684^**^	−0.0349	0.282^***^	0.000779	0.00289	0.0298	−0.0373
	(0.103)	(0.0314)	(0.0249)	(0.0756)	(0.0595)	(0.0371)	(0.0283)	(0.0312)
Claims	0.0129^*^	−0.00548^**^	0.00151	0.00975^**^	−0.0101	0.00681^**^	0.00394^**^	0.0120^***^
	(0.00690)	(0.00231)	(0.00131)	(0.00449)	(0.00694)	(0.00334)	(0.00172)	(0.00219)
Pages	0.00524^***^	0.0209^***^	0.00131^***^	0.0184^***^	0.0205^***^	0.0114^***^	0.00788^***^	0.00611^***^
	(0.00165)	(0.00159)	(0.000253)	(0.00394)	(0.00416)	(0.00198)	(0.000714)	(0.000966)
Constant	0.132^*^	0.253^***^	0.695^***^	−0.00797	0.112^**^	0.445^***^	0.522^***^	0.830^***^
	(0.0788)	(0.0301)	(0.0191)	(0.0784)	(0.0564)	(0.0359)	(0.0254)	(0.0244)
Observations	1,006	11,495	19,106	1,673	3,125	6,644	13,393	10,860
Pseudo *R*^2^	0.00895	0.00786	0.000854	0.0117	0.00311	0.00235	0.00438	0.00296

Standard errors in parentheses.

^***^p < 0.01.

^**^p < 0.05.

^*^p < 0.1.

**Table 10:** Summarizing variables.

**IPC sections**		**(1)**	**(2)**	**(3)**	**(4)**	**(5)**
	**Variables**	***N***	**Mean**	***SD***	**Min**	**Max**
A	Pages	1,006	20.21	29.19	3	498
	Claims	1,006	6.737	6.007	1	61
	F_Citations	1,006	1.611	2.404	0	27
	Neighbor	1,006	0.274	0.446	0	1
	Distant	1,006	0.229	0.420	0	1
B	Pages	11,495	15.94	9.795	3	220
	Claims	11,495	6.899	5.946	1	112
	F_Citations	11,495	2.006	2.843	0	53
	Neighbor	11,495	0.397	0.489	0	1
	Distant	11,495	0.266	0.442	0	1
C	Pages	19,106	28.72	41.99	3	1,012
	Claims	19,106	9.154	8.126	1	115
	F_Citations	19,106	2.151	3.115	0	55
	Neighbor	19,106	0.387	0.487	0	1
	Distant	19,106	0.245	0.430	0	1
D	Pages	1,673	16.53	9.446	4	105
	Claims	1,673	7.155	7.065	1	79
	F_Citations	1,673	1.791	2.533	0	26
	Neighbor	1,673	0.273	0.445	0	1
	Distant	1,673	0.296	0.457	0	1
E	Pages	3,125	12.71	6.963	3	132
	Claims	3,125	5.060	4.014	1	45
	F_Citations	3,125	1.420	1.958	0	27
	Neighbor	3,125	0.196	0.397	0	1
	Distant	3,125	0.204	0.403	0	1
F	Pages	6,644	15.53	8.935	3	177
	Claims	6,644	5.983	4.877	1	70
	F_Citations	6,644	1.972	2.503	0	26
	Neighbor	6,644	0.223	0.416	0	1
	Distant	6,644	0.222	0.416	0	1
G	Pages	13,393	22.52	20.54	3	932
	Claims	13,393	8.759	7.152	1	128
	F_Citations	13,393	2.262	3.854	0	254
	Neighbor	13,393	0.350	0.477	0	1
	Distant	13,393	0.258	0.438	0	1
H	Pages	10,860	18.34	16.67	3	600
	Claims	10,860	7.638	6.111	1	87
	F_Citations	10,860	2.890	3.943	0	56
	Neighbor	10,860	0.233	0.423	0	1
	Distant	10,860	0.190	0.392	0	1

**Figure 10:**
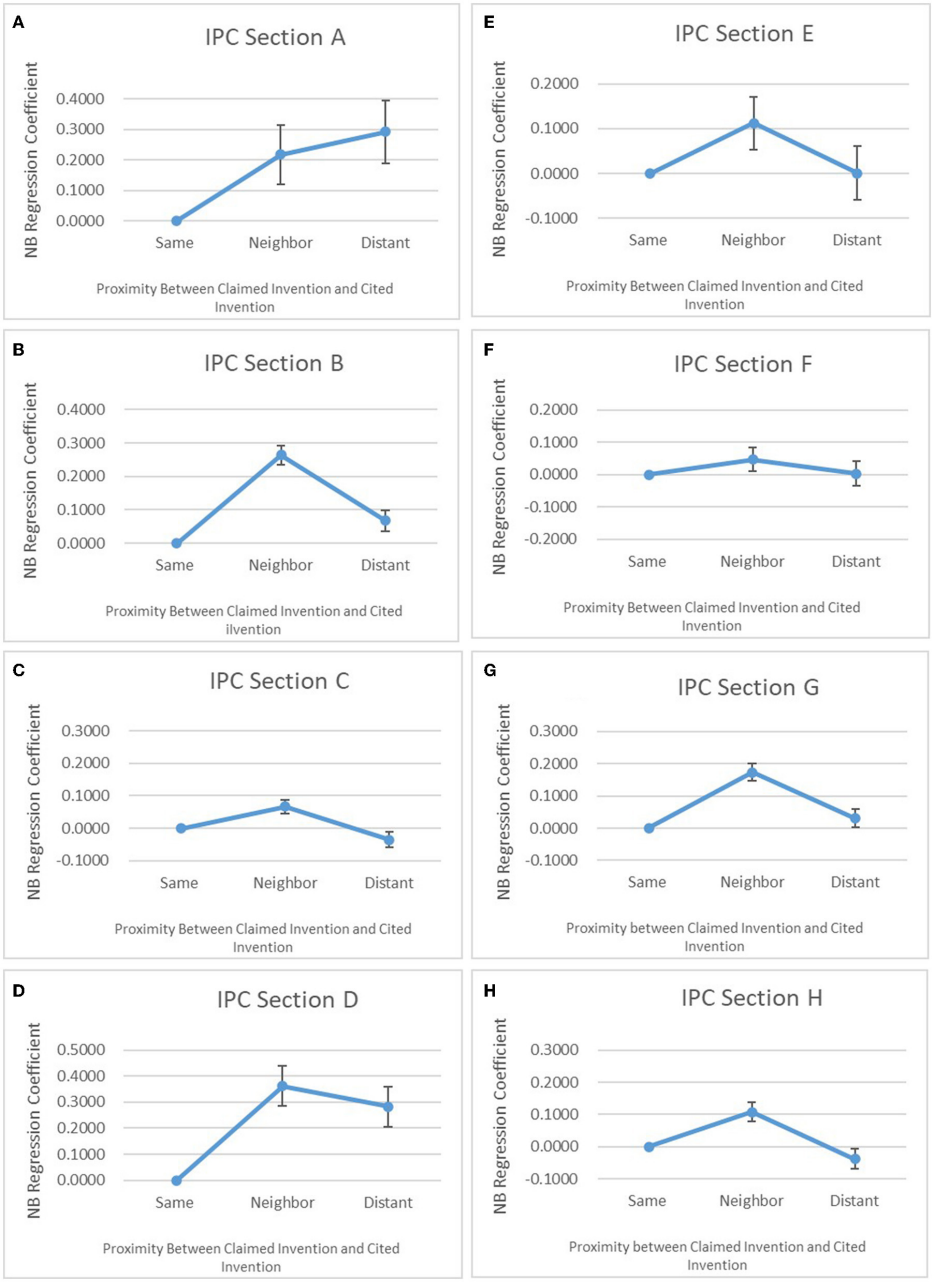
Graphs showing the relation between NB regression coefficient and proximity in each section of IPC with standard errors.

Figures 10A–H are graphs showing the results in Table 9. From these figures, it is understood that all IPC sections, except Section A, show the mountain-shaped pattern (Pattern II) that satisfies the hypothesis. Also, as mentioned earlier, the five IPC Sections B–D, G, and H have relatively small standard errors.

## Discussion

In this section, after reviewing the results of testing the hypothesis, the author examines the differences between the previous studies mentioned in Section Previous literature and the proposed technique.

### Results of testing the hypothesis presented in this article

The hypothesis was that there was a mountain-shaped relationship (an inverted-U-shaped relationship) between the patent proximity between claimed invention and cited invention and the impact of the subject application on subsequent applications. From the overall perspective of all patent applications filed in 2008 (refer Tables 4, 6; Figure 8), the hypothesis was supported. From the perspective of view of each IPC section, IPC Sections B–D, G, and H demonstrate the mountain-shaped pattern (Pattern II) that satisfies the hypothesis. The NB regression coefficients tend to increase as the technology field shifts from Same Field to Neighboring Field at the significance level of 1% or less for the abovementioned five sections of the IPC but tend to decrease as the technical field shifts from Neighboring to Distant Field (refer Table 9; Figures 10A–H).

The hypothesis was also tested for the dataset divided by the top 50 NetZero-related technical fields to analyze the impact of the subject application on subsequent applications (refer Table 8). As a subject of verification, it was found that there were 20 technical fields demonstrating the mountain-shaped pattern (Pattern II) that satisfies the hypothesis.

The NB Regression coefficient increases at the significance level of 10% or less as the technical field shifts from Same Field to Neighboring Field but decreases as the technical field shifts from (refer Nos. 3, 6, 9, 11–14, 16, 22, 26, 29, 31, 33, 35, 36, 42, 44, 45, 49, and 50 of Table 8; Figure 9B).

### Comparison with previous method

In considering the differences between the previous method mentioned in Section Previous literature and the technique proposed in this article, the author focuses on the study by Nemet (2012). This is because Nemet (2012, p. 1261) restricted the analysis to patents defined as energy technologies according to the list of energy classes suggested by Popp and Newell (2009). The energy technology patents analyzed by Nemet (2012) are included in the NetZero-related patent group analyzed in this article at a relatively high rate compared to the other two previous studies. From the comparison results of the search formulas shown in Appendix IV, it can be understood that many of the energy technology-related patents targeted by Nemet (2012) are included in the search conditions of this article.

It is understood that Nemet (2012) followed the same method as Nemet and Johnson (2012) and used only the primary classification and not the subsequent classifications. Nemet and Johnson (2012) coded each citation pair as “Far external,” “External,” and “Near” by comparing their classification at each level in the hierarchy. Since they allow the use of the IPC in place of the U.S. System (Nemet and Johnson, 2012, p. 14), IPC sections are used here as a superclass denoting “far external” and subclasses of the IPC as the converse denoting “near”. Therefore, using the analysis results of the technique proposed in this article, the author examines the difference in the results when analyzing using the IPC section and subsections.

### Comparison on “far external”

Tables 11A, B show the results of the subject patent application categorized into Distant Fields by the second filtering process (refer Figure 5). As shown in Table 2, the groups of citations cited in each application are shown in sets (Groups 1–6), in order of their citation, with up to six citations covered in this analysis. Results are shown for each of the six groups.

**Table 11:** Result of subject application categorized into distant fields.

**(A)**										
**Categories**	**Comparison of the subject patent application with each citation**		**Citations cited by the subject patent application**						**Total**	**Pct. (%)**
	**Between first classifications**	**Subsequent classifications of citations**	**Group 1**	**Group 2**	**Group 3**	**Group 4**	**Group 5**	**Group 6**		
Distant field	Non-coincidence		3,554	4,365	4,028	3,419	2,723	1,978	20,067	78
	Coincidence	Non-considered	1,085	1,210	1,093	896	697	524	5,505	22
	Total		4,639	5,575	5,121	4,315	3,420	2,502	25,572	100
**(B)**										
**Categories**	**Between first classifications**	**Subsequent classifications of citations**	**Citations cited by the subject patent application**						**Total**	**Pct. (%)**
			**Group 1**	**Group 2**	**Group 3**	**Group 4**	**Group 5**	**Group 6**		
Distant field	Non-coincidence		3,008	3,658	3,336	2,879	2,297	1,650	16,828	66
	Coincidence	Considered	1,631	1,917	1,785	1,436	1,123	852	8,744	34
	Total		4,639	5,575	5,121	4,315	3,420	2,502	25,572	100

A patent application categorized into Distant Fields must have a citation whose ATC classification is different from that of the subject application and whose IPC subclasses do not match any of the IPC subclasses of the subject application. For patent applications satisfying this condition, the author searched for citations whose IPC section of the first classification showed a match between the subject application and the citations. As a result, it turned out that about 22% corresponded as shown in Table 11A. This is mainly due to the differences in the hierarchical structure and breadth of classification between the ATC with 33 classifications and the IPC sections with 8 classifications. Next, as a result of conducting a search that included the subsequent classifications of the citations, the matching rate of the IPC sections increased to 34% as shown in Table 11B. This is obviously due to considering subsequent classifications as well as the first classifications. This search result reveals that the superclass denoting the “far external” category of Nemet (2012) partially overlaps Neighboring Fields as well as Distant Fields defined in the proposed technique.

### Comparison with “near”

Tables 12A, B show the result of the subject patent application categorized into Neighboring Fields by the second filtering process (refer Figure 5). As shown in Table 2, the citations for each application are shown divided into the six groups covered in this analysis.

**Table 12:** Result of subject application categorized into neighboring fields.

**(A)**										
**Categories**	**Comparison of the subject patent application with each citation**		**Citations cited by the subject patent application**						**Total**	**Pct. (%)**
	**Between first classifications**	**Subsequent classifications of citations**	**Group 1**	**Group 2**	**Group 3**	**Group 4**	**Group 5**	**Group 6**		
Neighboring field	Non-coincidence		12,497	12,143	10,892	9,016	6,857	5,162	56,567	99
	Coincidence	Non-considered	147	98	84	75	49	0	453	1
	Total		12,644	12,241	10,976	9,091	6,906	5,162	57,020	100
**(B)**										
**Categories**	**Between first classifications**	**Subsequent classifications of citations**	**Citations cited by the subject patent application**						**Total**	**Pct. (%)**
			**Group 1**	**Group 2**	**Group 3**	**Group 4**	**Group 5**	**Group 6**		
Neighboring field	Non-coincidence		4,683	4,693	4,118	3,469	2,687	1,940	21,590	38
	Coincidence	Considered	7,961	7,548	6,858	5,622	4,219	3,222	35,430	62
	Total		12,644	12,241	10,976	9,091	6,906	5,162	57,020	100

A patent application categorized into Neighboring Fields must have a citation whose ATC classification is different from that of the subject application, but at least one of whose IPC subclasses matches any of the IPC subclasses of the subject application. For a group of six citations satisfying this condition, the author searched for citations whose IPC subsection of the first classification showed a match between the subject application and the citations. As a result, it turned out that only 1% corresponded, as shown in Table 12A. Next, as a result of conducting a search that included the subsequent classifications of the citations, the matching rate of the IPC subsection increased from 1 to 62%, as shown in Table 12B. This is obviously due to considering subsequent classifications as well as the first classifications. This search result reveals that the IPC subclasses denoting the “near” category of Nemet (2012) partially overlap Same Field as well as Neighboring Fields defined in the proposed technique.

### Potential impact on analytical results

From the abovementioned two comparison results, it is considered that the superclass denoting “far external” and IPC subclasses denoting “near” shown in Nemet (2012) are shifted toward the Same Field side more than the Distant Fields and Neighboring Fields defined in this proposal. This becomes clear by considering the subsequent classifications in the comparison, although it is not clear from the comparison of the first classifications alone.

Unlike Nemet and Johnson (2012) and Nemet (2012) showed that for energy technologies, the integration of technologically distant prior art (i.e., distant knowledge) has a stronger positive effect on knowledge flows than the integration of technologically near prior art (i.e., local knowledge). However, considering the possibility that the analysis results will shift from the distant prior art to the near prior art, there is a possibility that the results of Nemet (2012) will be higher in the Neighboring Fields than in the Distant Fields, as in the analysis results of this proposal.

### Examination of backward citations excluded from analysis

**Figure 11:**
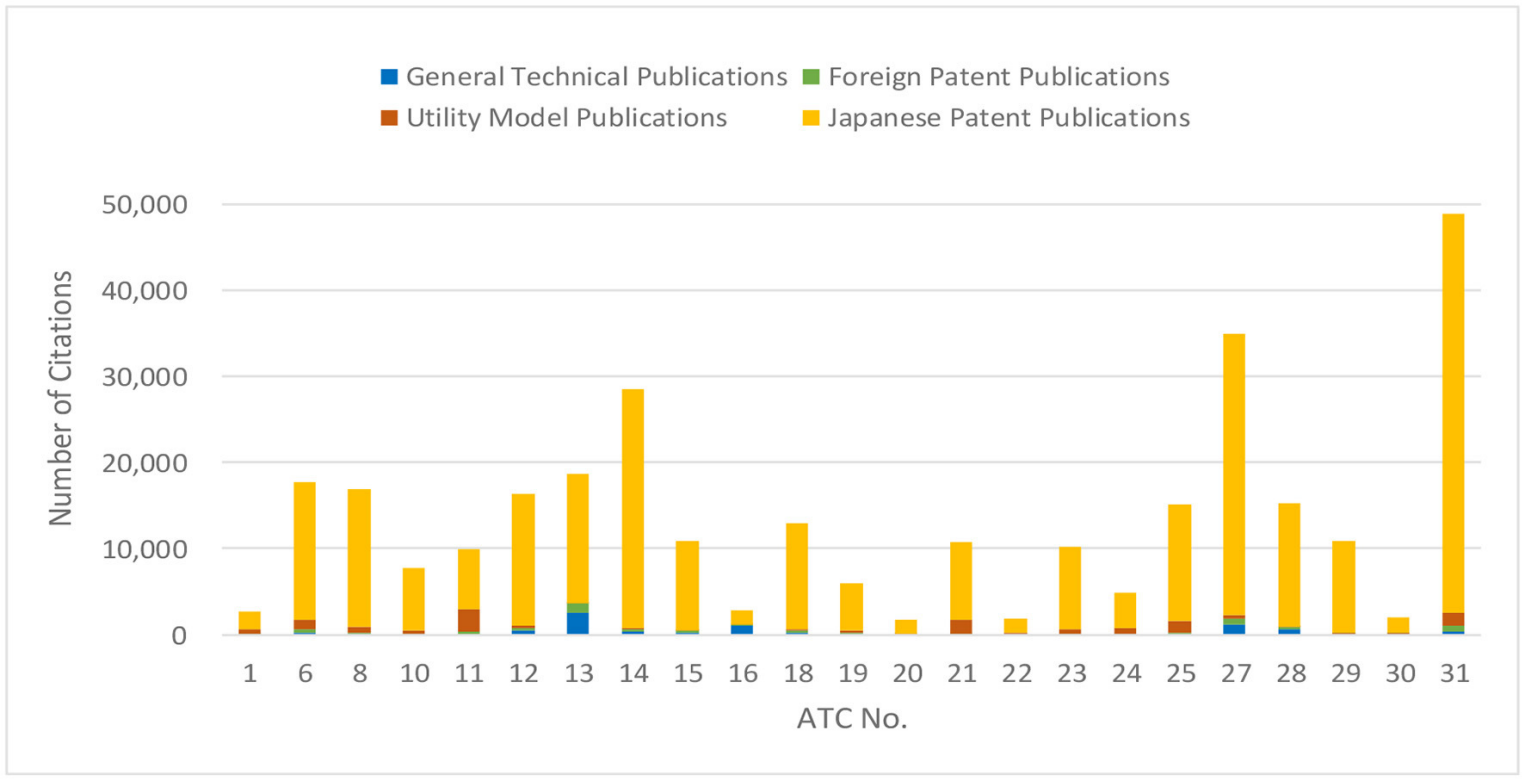
Bar graphs showing the percentage of each publication by ATC.

As mentioned in Section Data, this study does not analyze utility models, general technical documents, and foreign patent documents as backward citations. This subsection examines the proportion of these non-targeted publications in each technical field. Figure 11 is bar graphs showing the proportion of these three types of non-targeted publications to the total for each ATC field. From these graphs, it can be seen that in some technical fields, the proportion of non-targeted publications to the total is relatively high. For example, in ATC 13 (organic chemistry) and ATC 16 (biotechnology), the proportion of citing general technical publications is comparatively high, reaching 10 and 29%, respectively. In ATC 13 (organic chemistry) and ATC 16 (biotechnology), the proportion of citing general technical publications is comparatively high, reaching 10 and 29%, respectively. In addition, in ATC 1 (agriculture), ATC 11 (container and wrapping), and ATC 21 (construction), the proportion of citing utility model patent publications is high, reaching 16, 19, and 10%, respectively. Therefore, the author holds off on drawing conclusions about the results of analysis in these five technical fields.

## Conclusion

### Summary

This study showcases a technique to categorize NetZero-related patent applications into three technical fields according to the degree of proximity between claimed invention and cited inventions by comparing technological classifications between the patent applications and the cited applications thereof. In showing this method, the author explains the modified technique proposed by the author as well as the existing methods used in previous studies.

The proposed technique is different from those of previous studies in that it is characterized by comparing the technical fields of not only the primary classification but also the subsequent classifications. This is made possible by using two patent classifications without having a specific classification corresponding to the middle hierarchy in between, rather than using three patent classifications with different hierarchies. This technique reduces the possibility that two applications, even if they are the same in their subsequent classification, will be judged as applications in different technical fields because they are in different classes in the primary classification.

Using the proposed technique, the author examined the impact on the subsequent patent application of NetZero-related patent applications filed in Japan. As a result of the analysis, the author found that approximately 33% of subject applications, whose technical field differs from the backward citations when comparing the primary classification only, match one of the subsequent classifications when comparing them in consideration of the subsequent classifications as well. The author then found that the 33% of subject applications had a greater impact on the subsequent patent applications than the remaining applications. The evaluation results show that, looking at the top 50 NetZero-related technical fields as a whole, IPC Sections B–D, G, and H demonstrate the mountain-shaped pattern (Pattern II) that satisfies the hypothesis. They show that the NB Regression coefficient increases as the technical field shifts from the Same Field to the Neighboring Field at the significance level of 1% or less but decreases as the technical field shifts from the Neighboring to Distant Field.

### Practical implications

Japanese patent applications filed in 2008 were selected for this study for two reasons explained in Section Data and method for analysis. However, it is noted that 2008 was only selected for the initial analysis. There was a need to ensure sufficient time to obtain the backward citations necessary to measure the proximities of the patents and the forward citations necessary to measure the impact on the subsequent applications. The author believes that the trend of a mountain-shaped pattern that was obtained by this study will have a certain degree of continuity even if the patent filing year changes. Nevertheless, the need for multi-year surveys to supplement this single-year survey will be required.

Using the technique proposed in this article, an R&D manager can obtain information to consider whether to search distant technologies, neighboring technologies, or combinations within the same fields in terms of their impact on forward citations in that field of interest. According to the Japan Patent Office Annual Report published in 2022, 72% of patent applications filed in 2015 have already been requested for examination. Considering that it is possible to apply for accelerated examination for the target of green innovation, it is thought that a considerable number of backward citations have been accumulated even in patent applications filed in 2015.

The author believes that the proposed technique will be useful as support for an R&D manager in identifying/discovering technical fields to tackle the target issue in line with the COP 26 initiatives.

### Contribution of this article

In modern society, where technology is becoming more and more complicated, it is difficult to define technology with one technological classification. An increasing number of patent applications are matching subsequent classifications, even though the two patents have different initial classifications.

This study makes contributions to the previous literature by showing the proposed technique, which makes it possible to reduce the possibility that two inventions common to any of the subsequent classifications cannot be distinguished even if the primary classifications are different.

### Limitations

In this study, only Japanese patent publications are targeted as backward citations, and utility model patent publications, general technical publications, and foreign patent publications are not included in the analysis. However, since there are technical fields in which these non-targeted publications account for a relatively high percentage of backward citations, the author withholds conclusions about the five technical fields mentioned in the previous section. Furthermore, in this article, the author defined three technical fields, namely, “same,” “neighboring,” and “distant” in Section Hypothesis. However, it cannot be said that the definitions of these terms have been sufficiently defined and unified in comparison with multiple previous studies. These points remain to be addressed in a future study.

### Future research

This study was conducted for patent applications filed in 2008 only. There is still room to examine the hypothesis for patent applications filed in a year other than 2008. The author believes that the proposed technique can also be applied to the analysis of foreign patent applications. However, since there are important differences across jurisdictions in citation rules and practice as mentioned by Jaffe (2017, p. 1360), it is necessary to pay close attention to these differences when applying it.^12^

## Data availability statement

The original contributions presented in the study are included in the article/Supplementary material, further inquiries can be directed to the corresponding author.

## Author contributions

The author confirms being the sole contributor of this work and has approved it for publication.

## Footnotes

^1^The keywords used in the search are as follows. The actual search formula was prepared using Japanese keywords. “carbon neutral + global warming + de-carbonization + de-carbonizing + de-plasticization + COP26 + United Nations Framework Convention on Climate Change + net-zero emission + zero carbon emissions”.

^2^Although the annual number of patent application filed in Japan has remained at a high level of around 400,000 until 2008, the number of total patent applications in Japan decreased by 10.8% from 2008 to 2009 and has been gradually decreasing thereafter (see, e.g., JPO Annual Report, 2012, Part 1, p12, Fig. 1-1-1; JPO Status Report, 2022, Part 1, Chapter 1, p3, Figs. 1-1-4). Fujii and Managi (2016, p. 15) pointed out that the main reason for this dramatic decline is the economic recession triggered by the collapse of Lehman Brothers in 2008.

^3^Schumpeter identified five cases of new combinations: “(1) The introduction of a new good—or of a new quality of a good. (2) The introduction of a new method of production… (3) The opening of new market… (4) The conquest of a new source of supply of raw materials or half manufactured goods… (5) The carrying out of new organization of any industry….” (Shumpeter, 1934, p. 66).

^4^For example, Walsh (1995) pointed out that knowledge structures can restrict opportunities to acquire new knowledge and limit an individual's ability to comprehend information. In addition, focusing on core capabilities, Leonard-Barton (1992) pointed out the existence of core rigidities behind them. Tripsas and Gavetti (2000) found that the beliefs and dominant logic that Polaroid has accumulated in the past have created structural inertia, which has become a factor that hinders adaptation to technological change.

^5^For example, Fujifilm Corporation, a manufacturer of cameras and film, entered the cosmetics field in 2006 by paying attention to the similarity between film and skin, both of which are composed of mainly collagen. Idemitsu Kosan applied technology that had been cultivated in petrochemicals to a light-emitting material for organic EL, realizing high-efficiency, long-life pure blue light emission (commonly known as “Idemitsu Blue”), which was considered the most difficult to develop at the time. In another case, Seiko Epson has redefined the inkjet technology accumulated in the printer business as a micro liquid process technology and entered the organic EL display business using industrial production technology. In addition, Nitto Denko has been working on the development of “transdermal medication patches”, which are medical formulations, by making use of its advanced technological capabilities related to “adhesion technology” cultivated with adhesives. All of these cases have in common that they strive to develop new technology in different fields by making use of their own strengths.

^6^Although the context is different, a similar indication was made by Thompson and Fox-Kean (2005) regarding Jaffe et al. (1993), which found a corresponding “control” patent issued in the same primary U.S. patent class as the citing patent.

^7^Similar examples are found in the case of “Insecticides/Herbicides (IPC's Class A01N)” classified as ATC No. 13 in combination with organic chemistry (IPC Class C07) as well as the case of Explosives (IPC Class C06), which is separated from Chemistry (IPC Section C) and transferred to ATC No. 26 (Weapons and Explosives).

^8^It may be necessary to separately create an ATC table corresponding to the data of the IPC subclass in order to perform the filtering process because the ATC Nos information has been excluded from the IIP Patent Database since the data were extracted on August 31, 2017 (for unknown reasons).

^9^The JPO Examination Guidelines state that if there is a prior art document that the applicant finds useful, such as when making amendments, the examiner may include it in the prior art information (see Guidelines, Part I?, Chapter 2, Section 2.3). Although it does not serve as a basis for rejecting the claimed invention, it is considered to be useful information for determining the category of subject applications.

^10^In the United States, there is a duty to disclose to the U.S. Patent and Trademark Office any known prior art that is material to the patentability of any claim of a pending U.S. patent application. Failure to disclose relevant prior art during the prosecution of a patent application may lead to the patent being unenforceable (Code of Federal Regulations, Title 37: 37 C.F.R 1.56).

^11^It has been reported that the reason for this is that in the United States, “applicant citation” and “examiner citation” had not been distinguished as citation information contained in patent gazettes until 2001 (Yasukawa, 2017, p. 74).

^12^Especially in the United States, in addition to the difference mentioned in Note 11, it has been pointed out that the citation tendency of U.S. patent examiners had been different from that of Japanese examiners since the application publication system was introduced in November 2000 (Yasukawa, 2016, 2017). In addition, it has been reported that the U.S. Patent Classification (UPC) lacks some uniformity in the scale of technology as an issue (Hall and Trajtenberg, 2004, p. 4).
