# Supplementary material for: Should an R&D manager refer to distant technical fields? The effectiveness of new combinations with knowledge from different technical fields through the quantitative analysis of patent data related to NetZero
Source: Front Res Metr Anal. 2023 Apr 11;8:978249. doi: 10.3389/frma.2023.978249 (PMC10128997; doi:10.3389/frma.2023.978249)
Supplement: Supplementary file 1 [file Data_Sheet_1.docx]

Appendix I: Table of Aggregated Technology Classification (“ATC”)

Source of References: The table above is based on Goto, A. and Motohashi, K. (2005). Institute of Intellectual Property (IIP) Forum, Vol.63 pp.46 and Goto, A. and Motohashi, K. (2007), Research Policy, Vol. 36, Issue 9, November, Table 1, p.1433.

Appendix Ⅱ: English Translation of Search Formula

The actual search formula was prepared using Japanese keywords. The following English translation was prepared to be as close to the original as possible, but it is undeniable that it may contain expressions that are slightly different from the original, such as word order and synonyms, due to differences in language mechanisms.

Description of search expression symbols:

a) Boolean Operators: Symbols “+” and “*” represent AND and OR, respectively.

b) Proximity Operators: e.g. the symbol "A5" searches for publications with no more than 5 characters between the preceding and following words. The symbol "w5" searches for patent publications in which the preceding and following words are within 5 characters and the words are arranged in order.

c) Prefix Search: Symbol "?" performs a prefix search by deleting the last character in the character string.

d) Parenthesis: The parenthesized symbol "( )" or "[ ]" indicates that the search expression in the parentheses is prioritized for searching. The parenthesis symbol "< >" indicates that the word is represented by one word in Japanese.


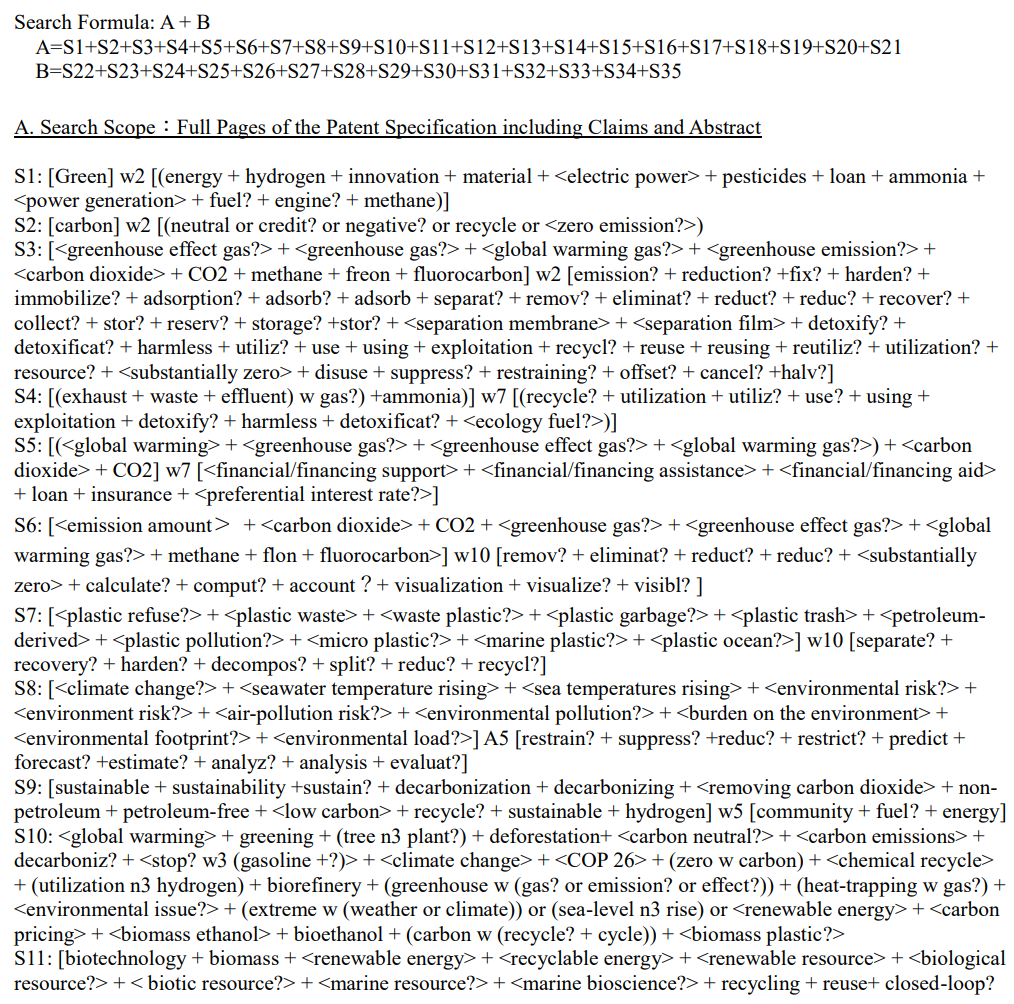


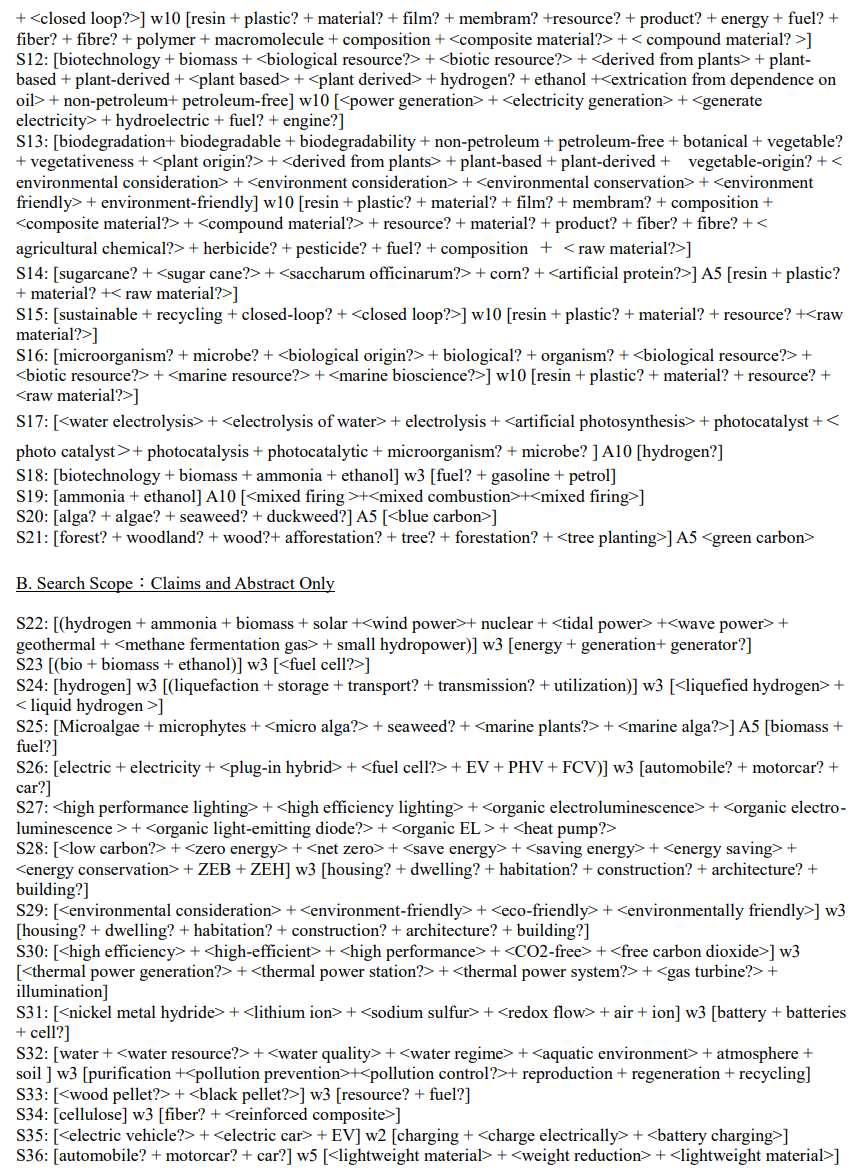


Appendix Ⅲ: Summary of Valuables used in the NB Regression analysis shown in Table 8

Nos. 35 to 50

Summary of Valuables


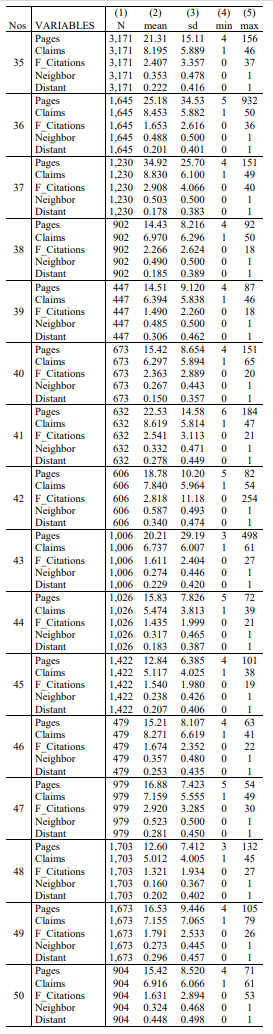


Nos. 1 to 17

Summary of Valuables


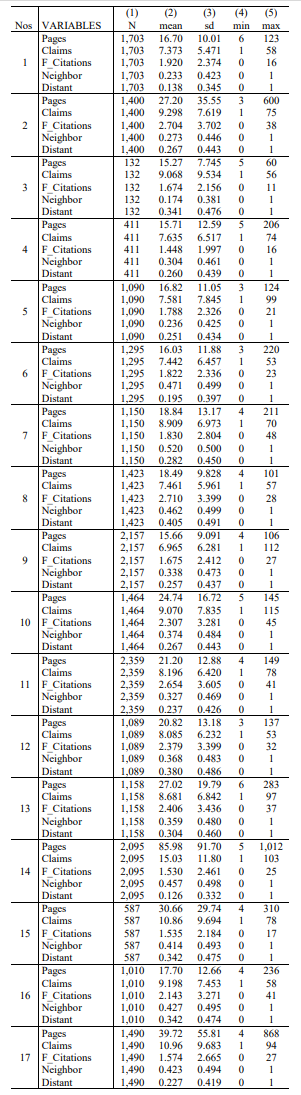


Nos. 18 to 34

Summary of Valuables


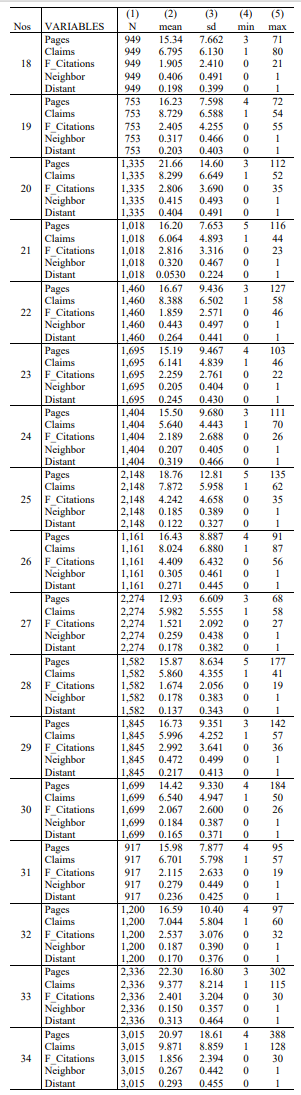


Appendix Ⅳ: Comparison with Nemet (2012)

As mentioned in Section 1, the following Table shows the relationship between research targets for searching energy technology related patents used in Nemet (2012) and the data retrieval formulas (see Appendix II) used to search for NetZero-related patents that are the subject of this paper's research.

From this comparison, it can be understood that many of the energy technology-related patents targeted by Nemet (2012) are included in the search conditions of this paper.

Table Showing the Relationship Between Research Targets for Nemet (2012) and the Data Retrieval Formulas (see Appendix II) Used in This Paper.


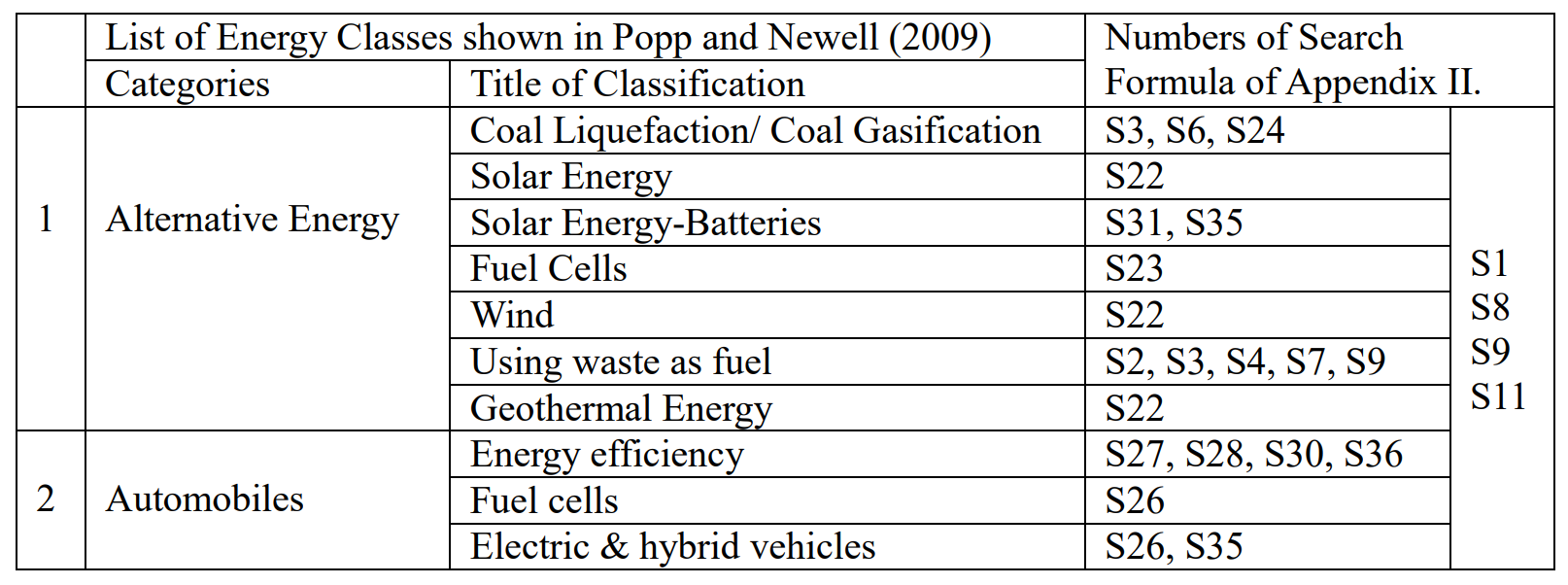


Appendix V: Examples of patent applications shown in the top 50 NetZero-related technical fields.

Following are examples of six representative technical fields selected from the top 50 NetZero-related technical fields, with 11 patent applications shown as examples.

a) Material Processing Laminate

Japanese patent applications s / n 2008-211081 is shown as an example of patent applications categorized in Same Field and belonging to B29C listed as No. 9 in Tables 1 and 8. This application provides a method for joining a resin and a metal with less environmental load by performing a dry surface treatment and joining them in a short time without using a surface treatment agent such as an acid, an alkali, or a primer treatment agent. This application was allowed as Patent 4,626,687 after overcoming the rejection, and was cited 13 times in the examination of subsequent patent applications.

b) Macromolecular Compounds,

　 Japanese patent application s/n 2008-81449 is shown as an example of patent applications categorized in Neighboring Field and belonging to C08J listed as No.12 in Tables 1 & 8. This application discloses a particle aggregation type monolithic organic porous material and a chemical filter, which have a high gas contaminant removing ability and are useful as ion exchangers for use in a deionized water production apparatus. This application was allowed as Patent No. 5,290,603 after overcoming the rejection, and was cited 32 times in the examination of subsequent applications. Another example categorized in Distant Field is Japanese Patent Application s/n 2008-123117. This invention relates to a fiber-reinforced thermoplastic resin composite material using a para-aramid fiber as a reinforcing material, which is light in weight and also high in strength, and is excellent in recyclability. This application was finally rejected because it did not meet the enablement and non-obvious requirements, but was cited 6 times in the examination of subsequent applications.

c) Inorganic Chemistry

Japanese patent application s/n 2008-22649 is shown as an example of patent applications categorized in Neighboring Fields and belonging to C01B shown as No.16 in Tables 1 & 8. This application discloses a CO_2_ recovery apparatus capable of being applied to a coal fired boiler and removing CO_2_ by bringing the exhaust gas containing CO_2_ into contact with the CO_2_ absorption liquid while suppressing the loss of a CO_2_-absorbing solution without lowering the concentration of the solution. This application was allowed as Patent No. 5,072,627 after overcoming the rejection, and was cited 10 times in the examination of subsequent applications. Further, Japanese patent application s/n 2010-500962 categorized in Neighboring Fields relates to hydrothermally stable microporous crystalline materials, which is able to reduce contaminants in exhaust gases contaminated with nitrogen oxides by the way of the selective catalytic reduction. Although this application was partially invalidated due to a trial for invalidation after patented, the application was cited 41 times in the examination of subsequent applications.

On the other hand, the patent application s/n 2008-186135 categorized in Distant Field discloses a method for producing a carbon microparticle from an organic raw material consisting mainly of lignin. While a large amount of lignin is contained in pulp waste liquid that is generated when wood chips are processed in a pulp mill, most of the lignin is currently incinerated. If it is possible to produce carbon microparticles from this pulp waste liquor, migration from fossil resources to biological resources and resources will be possible (Ibid. Paragraph 0043). The present application was allowed as Patent No. 5,062,593 without receiving any official rejections, and was cited 10 times in the examination of subsequent applications.

d) Organic Chemistry

　 Japanese patent application s/n 2008-58183 is shown as an example of patent applications categorized in Neighboring Fields and belonging to C07C shown as No.17 in Tables 1 & 8. This application discloses a method for regenerating an amine liquid, capable of effectively regenerating an ion exchange resin adsorbing a hardly decomposable component generated from a thermally stable amine salt, and capable of stably and efficiently carrying out a regeneration waste water treatment with high efficiency. This patent application was allowed as Patent No. 5,320,778 after overcoming the rejection, and was cited 8 times in the examination of subsequent applications.

e) Data Processing System for Supervision and Prediction Purposes

Japanese patent application s/n 2008-15375 is shown as an example of patent applications categorized in Distant Fields and belonging to G06Q shown as No.33 in Tables 1 & 8. This application discloses a charging system for an electric drive apparatus to give an incentive to the owner of the electric vehicle by evaluating how much exhaust gas is suppressed and adding points in accordance with the contribution to the environment. This patent application was allowed as Patent No. 5,152,644 after overcoming the rejection, and was cited 15 times in the examination of subsequent applications. Further, the patent application s/n 2008-109610 categorized in Distant Fields discloses a power management system which presents an energy saving plan conforming to an actual state of use of power and enables a resident to execute an energy saving action without difficulty. This patent application was allowed as Patent No. 5,123,039, and was cited 18 times in the examination of subsequent applications.

f) Greening Technology

　　Greening technology, consisting of IPC subclasses A01G (Agricultural & Marine Products), A01N (Insecticides and herbicides) and A01M (Pest Control), is listed as No.43 in Tables 1 & 8. Japanese patent application s/n 2008-284960 categorized in Distant Fields discloses an artificial alga bed construction capable of maintaining an effect of preventing the denuded seaweed bed (rocky-shore denudation) over a long period of time by sinking an iron-containing material such as steelmaking slag as a submersion material on the sea bottom and burying an iron acid elution unit in the backfilling part. The application was finally rejected as obvious because the claimed inventions could have been easily invented by a Skilled Person based on the combination of the cited documents. On the other hand, this patent application was cited 9 times in the examination of subsequent applications. Further, the patent application s/n 2008-153196 categorized in Distant Fields discloses a device for heating a greenhouse utilizing two heating systems comprising a heat pump system and a fuel burning system, and auxiliary utilizing a fuel burning-system heating device to improve heating efficiency of the heat pump-system heating device at low temperature. This patent application was allowed as Patent No. 5,203,807 after overcoming the rejection, and was cited 5 times in the examination of subsequent applications.
